# Geographically proximate rare species exhibit strong population divergence while maintaining intraspecific genetic diversity in *Homoranthus* (Myrtaceae)

**DOI:** 10.1093/aob/mcaf316

**Published:** 2025-12-09

**Authors:** Eilish S McMaster, Peter J Pemberton, Jeremy J Bruhl, Adam Fawcett, John T Hunter, Manu E Saunders, Elizabeth M Wandrag, Jia-Yee Samantha Yap, Ian R H Telford, Maurizio Rossetto, Rose L Andrew

**Affiliations:** School of Life and Environmental Sciences, University of Sydney, Camperdown, NSW 2050, Australia; Research Centre for Ecosystem Resilience, Botanic Gardens of Sydney, Sydney, NSW 2000, Australia; School of Environmental and Rural Sciences, University of New England, Armidale, NSW 2351, Australia; Botany and N.C.W. Beadle Herbarium, School of Environmental and Rural Science, University of New England, Armidale, NSW 2351, Australia; National Parks and Wildlife Service, New South Wales Department of Climate Change, Energy, the Environment and Water, Faulkner Street, Armidale, NSW 2350, Australia; School of Environmental and Rural Sciences, University of New England, Armidale, NSW 2351, Australia; School of Environmental and Rural Sciences, University of New England, Armidale, NSW 2351, Australia; Biological Sciences, School of Natural Sciences, University of Tasmania, Hobart, TAS 7001, Australia; Research Centre for Ecosystem Resilience, Botanic Gardens of Sydney, Sydney, NSW 2000, Australia; Botany and N.C.W. Beadle Herbarium, School of Environmental and Rural Science, University of New England, Armidale, NSW 2351, Australia; Research Centre for Ecosystem Resilience, Botanic Gardens of Sydney, Sydney, NSW 2000, Australia; School of Environmental and Rural Sciences, University of New England, Armidale, NSW 2351, Australia

**Keywords:** *Homoranthus A.Cunn. ex Schauer*, small range, narrow endemic, population genomics, conservation genomics, rock outcrop endemics, DArTseq, rare species

## Abstract

**Background and Aims:**

Species with small geographical ranges provide insights into adaptation, speciation and genetic drift, while also presenting clear conservation challenges. *Homoranthus* A.Cunn. ex Schauer (Myrtaceae), an Australian genus with many narrow endemics, offers a model for understanding how ecological and spatial factors drive diversification. We examined a regional hotspot with a high number of *Homoranthus* narrow endemics to assess patterns of genetic diversity and inform both evolutionary understanding and conservation planning.

**Methods:**

We generated genome-wide single-nucleotide polymorphism data using DArTseq for 282 individuals across 13 *Homoranthus* species (40 % of the genus), including 10 narrow endemics, to assess population genetic structure and diversity.

**Key Results:**

All species showed strong genetic isolation, even over a few kilometres, with populations diverging within hundreds of metres. *Homoranthus lunatus* includes two highly divergent, non-sister lineages, suggesting taxonomic revision. Inbreeding was common but unrelated to range size, and heterozygosity remained moderate, indicating intrinsic buffering of genetic diversity. Genome sizes were notably small relative to other angiosperms.

**Conclusions:**

Ecological isolation, life-history traits, and limited dispersal drive both speciation and extinction risk in *Homoranthus*. Diversification and endemism are linked to spatial isolation, highlighting the need for conservation strategies that address ecological connectivity in addition to species protection.

## INTRODUCTION

Species with restricted geographical distributions, i.e. narrow endemics, present unique challenges and opportunities in evolutionary biology and conservation science. Narrow endemics exist as small, isolated populations, which exacerbates genetic drift, inbreeding and the loss of genetic diversity, ultimately increasing the risk of extinction (Frankham *et al.*, 2017). These species are also vulnerable to stochastic disturbances (e.g. drought, fire or habitat alteration) that can rapidly jeopardize population viability (Ellstrand and Elam, 1993; Frankham, 1995, 1998). These taxa often have traits or processes to persist under demographic pressure, such as tolerance of inbreeding (Reinartz and Les, 1994; Razanajatovo *et al.*, 2019) or purging of deleterious alleles (Caballero *et al.*, 2017; Kleinman-Ruiz *et al.*, 2022). Understanding the evolutionary processes that lead to the emergence of these species and enable their survival is informative for conservation and for refining our models of population persistence.

Rocky outcrop species represent a particularly interesting subset of narrow endemics that are confined to the outcrop, its crevices, and the immediate surrounds, and many *Homoranthus* fall into this category (Hunter, 2003*a*). Granite and sandstone outcrops often form ‘islands’ of suitable habitat embedded within broader landscapes, acting as evolutionary crucibles that promote isolation, genetic drift, and local adaptation (Hopper, 2009; Hopper *et al.*, 2021). The nutrient-poor soils and microclimatic extremes on rocky outcrops restrict colonization and create spatially discrete niches; over evolutionary time, these conditions can drive parallel diversification and high levels of endemism (Baskin and Baskin, 1988; Ferris *et al.*, 2014; Pinheiro *et al.*, 2014). The isolating effect is particularly pronounced in entomophilous, obligate-seeding species that are pollinated by invertebrates with restricted foraging ranges, which further reduces connectivity between populations (Hopper, 2009). Consequently, rocky outcrop species often exhibit exceptionally narrow ranges and patchy distributions, which, in turn, elevate their extinction risk (Leão *et al.*, 2014; Nyberg *et al.*, 2025).

The Australian genus *Homoranthus* A.Cunn. ex Schauer (Myrtaceae) is one of the most range-restricted plant groups in the continent, with many species confined to tiny patches and formally listed as threatened. Comprising 32 species endemic to mainland Australia, it is notable for the unusually high density of narrow endemics occurring in close geographical proximity (Craven and Jones, 1991; Copeland *et al.*, 2011). Although some species, such as *Homoranthus virgatus* and *Homoranthus flavescens*, have relatively broad distributions (area of occurrence > 1000 km^2^), most species of *Homoranthus* are naturally rare (Craven and Jones, 1991; Copeland *et al.*, 2011) and are listed as threatened under state or federal legislation (Briggs and Leigh, 1996; Hunter, 1998; Bean, 2000, 2009). Many, but not all, narrow endemic species of *Homoranthus* are confined to rocky outcrops: some, such as *Homoranthus bebo* and *Homoranthus vagans*, occupy deep sandy soils in open forest (Copeland *et al.*, 2011). This ecological variation makes *Homoranthus* a particularly valuable genus for examining the roles of substrate specificity, geographical isolation and life-history traits in driving divergence.

Despite its conservation significance and ecological intrigue, the evolutionary relationships and genetic diversity of *Homoranthus* species remain poorly understood. Previous research has focused primarily on morphological and cytological traits (Craven and Jones, 1991; Copeland *et al.*, 2011), leaving open questions about how speciation, genetic drift and historical connectivity have shaped diversity within and among species.

In this study, we applied a multispecies population genomics approach to investigate the genetic structure, diversity and gene flow across 13 species of *Homoranthus* from the Queensland (QLD) and New South Wales (NSW) border region, which is a hotspot for rare species of *Homoranthus*. Ten of the study species are endemics with highly restricted distributions, mostly associated with rock outcrops, and are listed as threatened at the state or federal level (Table 1). We contrast these with three more widespread congeners, two of which are also listed as Vulnerable in Queensland. By sampling at the population level across species with varying threat status, we aim to assess whether range restriction is correlated with genetic diversity and inbreeding; assess connectivity among disjunct populations; and evaluate whether these genomic patterns can inform conservation strategies tailored to the specific ecological and evolutionary contexts of narrow endemic taxa.

**Table 1.** *Homoranthus* species included in this study, showing conservation status, collection sites and sample sizes.

Species	Growth form/habitat/reserved	Federal	NSW	QLD	Collection site	*n*
*H. bebo* L.M.Copel.^a^	Decumbent. Open Forest. Deep sandy soils. Protected land.	CR	CR		Camp Creek East	46
					Camp Creek West	8
					NSW Northwestern Plains (Herbarium)	1
*H. binghiensis* J.T.Hunter^a^	Erect. Outcrop surrounds and crevices. Protected land.		EN		Torrington Site 2 (T2)	22
					Torrington Site 5 (T5)	25
*H. bruhlii* L.M.Copel.^a^	Erect. Outcrop surrounds and crevices. Private land.	CR	CR		Tenterfield	5
*H. croftianus* J.T.Hunter^a^	Erect. Outcrop surrounds and crevices. Protected land.		CR		Bolivia Site 1 (B1)	29
					Bolivia Site 2 (B2)	39
*H. darwinioides* (Maiden & Betche) Cheel	Ascending. Deep sandy soils. Protected and private land.	VU	VU		NSW Central Western Slopes (Herbarium)	3
*H. inopinatus* L.M.Copel., J.Holmes & G.Holmes^a^	Erect. Deeper outcrop surrounds and crevices. Private land.			CR	Ballandean	16
*H. lunatus* Craven & S.R.Jones^ab^	Ascending. Outcrop surrounds and crevices. Protected land.	VU	VU		Boonoo Boonoo (BB) Cypress	9
					Boonoo Boonoo (BB) Morgan	14
					Basket Swamp (BS)	9
					Torrington Site 3 (T3)^b^	11
*H. melanostictus* Craven & S.R.Jones	Ascending. Widely distributed in woodlands on sandy soils. Protected and private land.				Barakula State Forest	1
					Waroonga State Forest	1
*H. montanus* Craven & S.R.Jones^a^	Erect. Deeper outcrop surrounds and crevices. Protected land.	VU		CR	Hillview Nature Reserve	13
*H. papillatus* Byrnes^a^	Ascending. Outcrop surrounds and crevices. Protected land.			CR	Mt Norman	11
*H. prolixus* Craven & S.R.Jones	Ascending (mostly). Outcrop surrounds and crevices. Protected and private land.	VU	VU		Ironbark	4
*H. vagans* L.M.Copel.^a^	Decumbent. Open Forest. Deep sandy soils. Protected land.			VU	Wundal Range National Park	15

The number of individuals sampled at each site (*n*) is provided. Conservation status is reported at both federal and state levels: Commonwealth listings under the Environment Protection and Biodiversity Conservation Act 1999 (EPBC), New South Wales under the Biodiversity Conservation Act 2016 (BC), and Queensland under the Nature Conservation Act 1992 (NC). Abbreviations for listing status are CR (Critically Endangered), EN (Endangered) and VU (Vulnerable). Growth form/habitat/reserved identifies which of the three forms the species has, brief habitat characteristics, and whether the species exists on protected and/or private land.

^a^Range-restricted species.

^b^The samples collected as *H. lunatus* from Torrington Site 3 were determined to be an undescribed genetically and morphologically distinct species referred to throughout as *Homoranthus* sp. Torrington (formal description in review).

## MATERIALS AND METHODS

### Study area and species

**Fig. 1.**
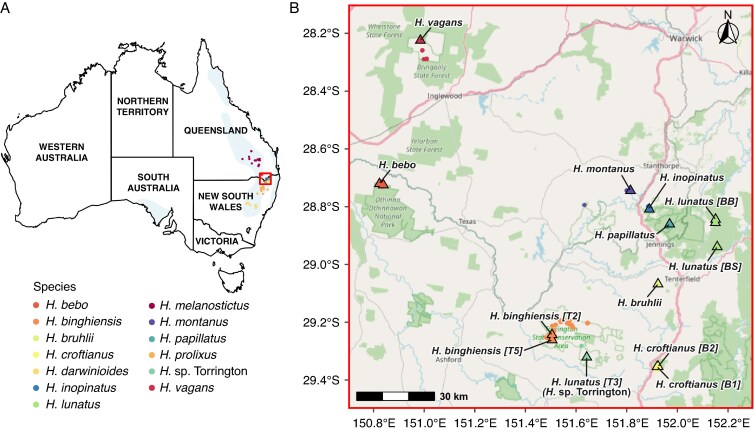
Geographical distribution of *Homoranthus* species in Australia and sampling locations of individuals genotyped in this study. (A) Blue-shaded areas show the known distribution of *Homoranthus* species across Australia, with the study region highlighted by a red box. Points indicate observations from the Atlas of Living Australia (ALA; since 2000) of species included in this study. (B) Detailed view of the study area, showing collection sites for each species as coloured points. Observations are shown as circles, and sampling sites collected in this study are shown as triangles. Populations are labelled by species and site abbreviation where appropriate (see Table 1). *Homoranthus darwinioides*, *H. melanostictus*, and *H. prolixus* were sampled outside the study area and are not shown. The Torrington population is referred to here as *Homoranthus* sp. Torrington (previously considered part of *H. lunatus*). Map layer is OSM available as open data under the Open Data Commons Open Database License (Fellows *et al.*, 2023).

This study investigates rare and narrow endemic members of the genus *Homoranthus* (Myrtaceae) occurring in and around the Northern Tablelands of New South Wales, Australia. The primary study area extends 130 km north–south and 150 km east–west along the New South Wales—Queensland border, ∼140 km inland from the coast (Fig. 1). It encompasses parts of the New England Tablelands, Nandewar, and Brigalow Belt South IBRA bioregions (Department of Climate Change, 2012, the Environment and Water, 2012). The landscape is dominated by dry sclerophyll vegetation with numerous granite outcrops (State Government of NSW and NSW Department of Climate Change, Energy, the Environment and Water, 2012, 2020). Although there are many protected areas within the study area, much of the land has been modified for livestock grazing and agriculture. Annual rainfall declines from ∼900 mm in the east to ∼600 mm in the west.

Within the study area, ten *Homoranthus* species occur in highly localized and fragmented populations, typically restricted to fewer than three subpopulations: *H. bebo* L.M.Copel., *H. binghiensis* J.T.Hunter, *H. bruhlii* L.M.Copel., *H. croftianus* J.T.Hunter, *H. inopinatus* L.M.Copel., J.Holmes & G.Holmes, *H. lunatus* Craven & S.R.Jones, *H. montanus* Craven & S.R.Jones, *H. papillatus* Byrnes, *H. vagans* L.M.Copel., and a genetically and morphologically distinct population previously considered a disjunct subpopulation of *H. lunatus*, here referred to as *H.* sp. Torrington (not formally described; in review at Telopea ISSN: 0312-9764, 2200-4025). Minimum interspecific distances are ∼10 km (e.g. *H. inopinatus*, *H. montanus* and *H. papillatus*), and maximum separation is ∼130 km (between *H. bebo* and *H. croftianus*).

**Fig. 2.**
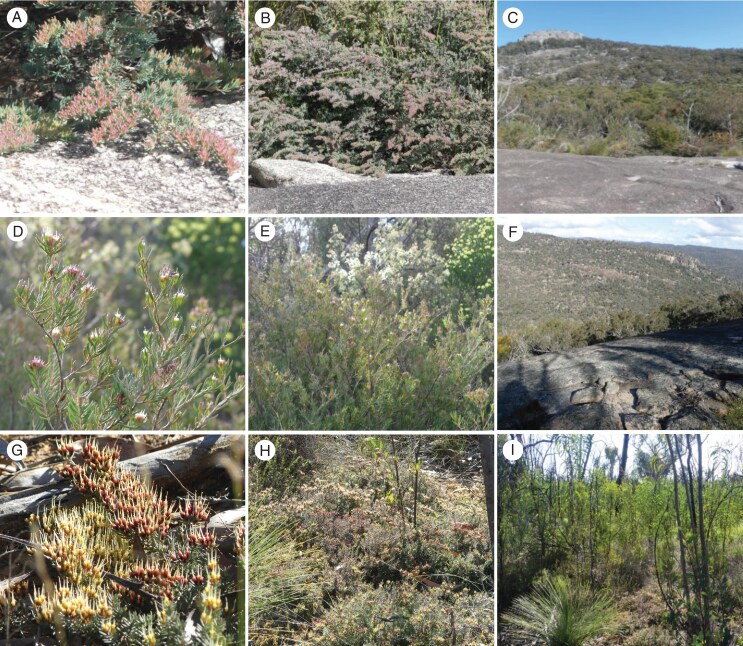
Flowering branchlets, growth forms and habitats of *Homoranthus*. (A) *Homoranthus papillatus* lower flower branchlet spreading over an outcrop surface. The multiple single flowers are yet to open, with red bracteoles (floral prophylls) visible. (B) Ascending form of *H. papillatus* (1.0 m) with layered branches. (C) *Homoranthus papillatus* is found in crevices and outcrop edges on the undulating slopes of Mt Norman, where rock sheets can provide limited fire protection. If obligate seeders are destroyed by fire, their seed banks may allow for rapid recruitment. (D) *Homoranthus inopinatus* erect flowering branchlets with three to six flowers on each branchlet. (E) The erect form of *H. inopinatus* (1.7 m) in the forefront of the ecocline between the rock sheet and taller vegetation. (F) The steep rock sheet below *H. inopinatus* provides a physical barrier to uphill fires. This species has been observed to resprout, but old-growth individuals (stems 50 mm diameter) indicate that the niche habitat has provided protection from several wildfires. (G) *Homoranthus vagans* flowering branchlets lying on the ground. Yellow flowers are new, turning red as they age. (H) *Homoranthus vagans* (0.2 m) is decumbent and forms a vegetive mat. (I) The habitat of *H. vagans* (foreground) is open woodland, with grass and shrub understorey. A low-intensity wildfire occurred in October 2023; the surrounding bush was burnt and is now in post-fire recovery. The *H. vagans* mats at this location survived unburnt and intact, possibly owing to its decumbent form.

*Homoranthus* are perennial shrubs, with most species having laterally compressed leaves (leaf sides are larger in area than the top of the leaf), and they have three distinct growth forms: erect, ascending or decumbent (Copeland *et al.*, 2011), with all three forms represented in our study (Fig. 2). Eight of our study species are outcrop endemics and are restricted to shallow, sandy soils (Table 1). The outcrop species often dominate plant communities in rock crevices or in the ecoclines between the rock sheet and the surrounding bush, where they are well placed to use moisture runoff from rock sheets. Outcrops have limited recruitment sites and below-ground resources, which favour obligate seeders that are long-lived, have a low turnover and monopolize outcrop resources (Hunter, 2003*b*).

Plant life history is largely unknown, with longevity suggested to exceed 30 years (Hunter 2002). Based on observations of *H.* sp. Torrington following the 2019 fires, where all but one adult plant was lost but where flowering individuals were recorded by 2024, the primary juvenile period is likely to be within 5 years (pers. obs. P. Pemberton 2024). However, the effective juvenile period, sufficient to restore the seed bank after disturbance, might extend several seasons longer. Although mass recruitment typically follows major disturbance events, such as fire, low-level recruitment might occur intermittently. Flowering in *Homoranthus* is generally seasonal, but owing to the extreme and variable conditions of outcrop habitats, sporadic flowering throughout the year is not uncommon and might represent a bet-hedging strategy to maximize reproductive success in unpredictable climatic conditions (Copeland *et al.*, 2011). Outcrop *Homoranthus* species are therefore likely to be obligate seeders that maintain persistent seed banks with a long dormancy (Saunders *et al.*, 2024), resulting in mass recruitment after disturbance events, such as bushfire.

Resprouting is common amongst outcrop flora (Hunter, 2003*a*), and resprouting was observed in two of our study outcrop species with an erect form, *H. inopinatus* (pers. obs. P. Pemberton 2024) and *H. montanus* (P. Donatiu pers. comm., September 2025). The remaining two study species are decumbent, form vegetation mats, exist on deeper soils in open woodlands and can root at branchlet nodes, allowing for the possibility of clonal propagation (Copeland *et al.*, 2011; Hunter, 2016; Le Breton and Auld, 2018). The elevational range of the study species is 300–400 m for decumbent species and 700–1000 m for outcrop species. All study species have chromosome numbers of 2*n* = 18, except *H. binghiensis* (2*n* = 20) (Copeland *et al.*, 2008); counts for *H. inopinatus* and *H.* sp. Torrington are unknown. Flowers are small, scented and produce large quantities of nectar, and these traits are generally attractive to insects (Wright and Schiestl, 2009; Erickson et al., 2022). The morphology of the flower, specifically the relative position of the corolla and reproductive organs (Dellinger, 2020), suggests that large-bodied insect visitors are necessary to effect pollen transfer. Invertebrates have been observed as floral visitors (e.g. native/non-native bees, butterflies, crane flies and ants; Saunders *et al.*, 2024), in addition to vertebrates, but the pollinators have not been confirmed. The spatial separation of the anther and stigma indicates avoidance of self-fertilization. Seed dispersal is expected to be local; myrmecochory is present in the closely related *Darwinia* species, but the seed dispersal distance is usually of the order of a few metres (Auld and Ooi, 2009).

Eight of the ten focal species occur within protected areas today, though many sites were historically affected by grazing, clearing or mining. Some species distributions have probably been reduced, e.g. *H. bebo* occurs on the boundary of a reserve adjoining cleared land (Hunter, 2015), and the Morgans Gully subpopulation of *H. lunatus* was affected by gravel washing during gold mining. Granite outcrop populations appear to have been partly buffered from past land-use impacts. Although the extent of historical range loss cannot be determined, most outcrop taxa probably experienced only limited effects from grazing or forestry. Many *Homoranthus* species exhibit internally disjunct subpopulations even in continuous undisturbed habitat, suggesting that natural fragmentation is common. The study species therefore probably had similar patchy distributions and rarity before European settlement, perhaps with some reduction in occurrence but not wholesale decline. All species considered in this study except *H. melanostictus* are listed as threatened at state or federal levels and are primarily at risk from habitat degradation attributable to grazing by feral animals (goats, pigs and rabbits), encroachment by invasive plant species, and inappropriate fire regimes (Department of Climate Change, Energy, the Environment and Water, Australian Government, 2008*a*, *b*, *c*, *d*; Le Breton, 2016; Phillips, 2016; Department of Climate Change, 2018, the Environment and Water, 2018).

### Sampling and sequencing

Leaf tissue samples were collected from wild populations of the nine target species of *Homoranthus* within the study area (Table 1; Fig. 1). Sampling aimed to be spatially and numerically representative, with individuals sampled per site depending on population size and accessibility. Individuals were spaced ≥5 m apart where possible to minimize the likelihood of sampling clones (Rossetto *et al.*, 2021).

To provide broader phylogenetic and biogeographical context, additional samples of *H. melanostictus* were collected from populations further north in Queensland, and *H. prolixus* was sampled from populations south of the study area. Three herbarium samples of *H. darwinioides*, originally collected from wild populations ∼50 km south of the study region, and one herbarium sample of *H. bebo* were sequenced from the National Herbarium of NSW (Table 1). These herbarium samples were included to increase taxonomic coverage and provide temporal context for comparison with current populations.

Approximately 3 g of leaf tissue was taken from each plant, with geographical coordinates recorded. Samples were preserved in silica gel until DNA extraction and stored in a cool room at 14 °C. Genotyping was performed using DArTseq, a reduced-representation sequencing method implemented by Diversity Arrays Technology Australia Pty Ltd. This approach involves digestion with a restriction enzyme, followed by Illumina short-read sequencing of the digested products, with single-nucleotide polymorphisms (SNPs) called using proprietary analytical pipelines (Jaccoud *et al.*, 2001; Kilian *et al.*, 2012). Genotype data were provided as SNP matrices with quality-informing statistics for each bi-allelic marker and sample. Two duplicate samples of *H. croftianus* and *H. lunatus* from Torrington were included as technical replicates (Table 1). Analyses were conducted in R v.4.3.1 (R Core Team, 2021) unless otherwise specified.

### Spatial analyses

Both area of occupancy (AOO) and extent of occurrence (EOO) are key measures of range size and are used in assessing species vulnerability to extinction by the International Union for Conservation of Nature (IUCN) (Brooks *et al.*, 2019). Under IUCN criteria, the EOO is the area contained within the shortest continuous boundary that encompasses all known occurrences, whereas the AOO is the area where the species is actually found, calculated by summing the area of all occupied 2 km × 2 km grid cells. These metrics were calculated to contextualize the rarity of species of *Homoranthus* and assess range size association with diversity analyses.

To quantify AOO and EOO in target species, Atlas of Living Australia (ALA) records were combined with locations of samples collected for this study. ALA records for target species since 2000 were retrieved using galah v2.1.1 (Westgate *et al*., 2025). Spatially suspect records were excluded from the analysis. AOO and EOO calculations followed the guidelines outlined by the IUCN, using the *red* v.1.6.1 (Cardoso, 2017) and *sf* v1.0-21 R packages (Pebesma, 2018) for spatial analysis and range size calculations. These results were then compared with the geographical range size data from Le Breton *et al.* (2019) to assess the range percentile of the target species among plants in NSW.

### Population genomics

#### Data filtering

Data filtering was performed using custom scripts implemented with RRtools v.0.1.0 (https://github.com/jasongbragg/RRtools). Loci with >80 % missing data were removed, as were fixed loci and loci with minimum reproducibility <96 %. Reproducibility measures the marker consistency across technical replicates; the retained loci averaged 99.2 %, indicating very high reliability. To reduce linkage disequilibrium, the dataset was further reduced to include only one SNP per DNA fragment. Samples with >80 % missing loci were excluded. Following these filtering steps, the dataset comprised 282 samples and 29 894 loci, which were used for subsequent analyses.

Herbarium samples (three *H. darwinioides*, one *H. bebo* and two *H. melanostictus*) showed variable data quality relative to the overall dataset. The *H. bebo* herbarium sample had missing data comparable to other *H. bebo* individuals (38.2 % vs. species mean 35.8 %). In contrast, *H. darwinioides* and *H. melanostictus* herbarium samples had higher missingness (57.4 % and 77.2 %, respectively) relative to the overall average (38.5 %), probably reflecting both poorer DNA quality and the small number of samples available for these species.

#### Genomic analyses

Dimensionality reduction was applied to assess genetic clustering. A biallelic genotype matrix was used to calculate pairwise Euclidean distances between samples with the dist function from the stats package v.4.3.1. The distance matrix was subjected to Barnes-Hut t-Distributed Stochastic Neighbor Embedding (t-SNE) using the Rtsne package v.0.17 (Krijthe, 2015). The analysis was conducted with the following parameters: dimensions = 2; perplexity = 18; and theta = 0. t-SNE was selected over principal component analysis for its superior performance in identifying clusters within data (Maaten and Hinton, 2008).

A phylogenetic network was also constructed from the pairwise distance matrix using fast-NNT v.0.2.1 in R (Newell and McMaster, 2025) implementing the SplitsTree4 v.4.18.2 algorithm (Huson, 1998; Huson and Bryant, 2006). The network was visualized using tanggle v.1.8.0 (https://github.com/KlausVigo/tanggle).

ADMIXTURE v.1.3.0 (Linux) was used to estimate ancestry proportions (Alexander and Lange, 2011). Prior to analysis, loci were filtered to retain only those with a minor allele frequency ≥ 1 %, resulting in 25 899 loci. To reduce potential bias from uneven sample sizes (Wang, 2017; Toyama *et al.*, 2020), the dataset was subset to include a maximum of ten individuals per species (five individuals per subpopulation for *H. binghiensis*, *H. croftianus* and *H. lunatus*, which have genetically distinct subpopulations). *Homoranthus melanostictus* was excluded altogether owing to small sample size and high missingness. Genotypes were exported to PLINK bed format using the snpgdsGDS2BED function in SNPRelate v.1.36.1 R package. ADMIXTURE was run initially in unsupervised mode on the subset dataset with *k* values ranging from 1 to 20, using 20-fold cross-validation to evaluate model fit. The allele frequency estimates (file P) from the best-supported *k* = 12 was then used as fixed input to project ancestry estimates onto the full dataset containing all samples.

Kinship within subpopulations was calculated using the method of moments (MoM) for Identity-By-Descent (IBD) (Purcell *et al.*, 2007; Chang *et al.*, 2015) implemented in the SNPRelate. A minor allele frequency filter of 5 % and a missingness threshold of 20 % were applied to ensure robust IBD estimation (McMaster *et al.*, 2025*b*). Duplicate clones, defined as pairs with kinship >1/2^3/2^ (Manichaikul *et al.*, 2010), were excluded in subsequent diversity statistics and pairwise fixation index (*F*_ST_) analyses.

Population differentiation was assessed by calculating *F*_ST_ between sites using the snpgdsFst function from the SNPRelate. The Weir and Hill (Weir and Hill, 2002) estimator was implemented for *F*_ST_ calculation. To ensure robust estimates, loci were filtered to allow a maximum of 30 % missing data, a minimum minor allele frequency of 5 % and a minimum site sample size of three individuals.

Genetic diversity statistics were calculated for each population using the *fastDiversity* package v.1.0.0 (Keenan *et al.*, 2013; McMaster, 2025). Clonal individuals were excluded prior to the genetic diversity analysis. Species with fewer than ten samples (*H. bruhlii*, *H. darwinioides*, *H. melanostictus*, and *H. prolixus*) were excluded. Within each species, loci were filtered to retain those with no more than 30 % missing data and a minimum minor allele frequency of 5 %. For each population, mean observed heterozygosity (*H*_O_), expected heterozygosity (*H*_E_) and the inbreeding coefficient (*F*_IS_) were estimated from 1000 bootstrap replicates, each based on random resampling of 500 loci with replacement (Goudet, 2005; Excoffier and Lischer, 2010). Both the mean and the 2.5 % and 97.5 % confidence intervals were derived from the bootstrap distributions. Hardy–Weinberg equilibrium was tested at each locus using HardyWeinberg v.1.7.9 (Graffelman *et al.*, 2025), and overall *P*-values were calculated using Fisher’s combined probability test.

Individual values for multilocus heterozygosity (MLH) and inbreeding coefficient (*f*) were also calculated within populations and used to examine the relationship between genetic diversity and range size. To test this, we fitted linear models with MLH and *f* as response variables and with EOO and species identity as predictors. Species with fewer than ten samples were excluded.

To prepare the DArTseq genotype data for phylogenetic analysis in RAxML with ascertainment bias correction, loci lacking at least one homozygous representative for each allele (e.g. 0/1 or 1/2), which RAxML treats as invariant, were identified and removed. This filtering reduced the dataset to 29 237 loci used for tree inference. Genotypes encoded as alternative allele counts (0, 1, 2) were then converted into nucleotide characters using the provided reference and alternative alleles. Homozygous reference genotypes were coded as the reference nucleotide, homozygous alternative genotypes as the alternative nucleotide, and heterozygous genotypes were encoded with appropriate International Union of Pure and Applied Chemistry (IUPAC) ambiguity codes to represent both alleles. The resulting character matrix was exported as a FASTA file using the ape package v.5.8-1 (Paradis *et al.*, 2004).

A maximum likelihood phylogenetic tree was estimated using RAxML-NG (Stamatakis, 2014; Kozlov *et al.*, 2019) on the ACCESS computational platform hosted on the CIPRES Science Gateway (Miller *et al.*, 2010). The analysis incorporated the Lewis ascertainment bias correction to account for presence/absence bias in the dataset. A Gamma model of rate heterogeneity (+G) was applied to account for variation in substitution rates across sites. The tree inference was performed using 100 bootstrap replicates [transfer bootstrap expectation (TBE); Zaharias *et al.*, 2023], using the default Rapid Hill Climbing algorithm for tree search optimization (Weigert *et al.*, 2014; Melville *et al.*, 2017; Lal *et al.*, 2018; Fahey *et al.*, 2022). The final tree was visualized using ggtree v.3.10.0 (Yu *et al.*, 2017).

Additional packages used for analysis and plotting include data.table v.1.17.4 (Barrett *et al.*, 2025), dplyr v.1.1.4 (Wickham *et al.*, 2023), ggplot2 v.3.5.1 (Wickham, 2016), ggthemes v.5.0.0 (Arnold, 2024), RColorBrewer v.1.1-3 (Neuwirth, 2022), ComplexHeatmap v.2.18.0 (Gu, 2022), magrittr v.2.0.3 (Milton Bache *et al.*, 2022), and ggpubr v.0.6.0 (Kassambara, 2023).

#### Genome size estimation

Genome size was estimated using flow cytometry to investigate potential cytogenetic changes associated with speciation, because variation in genome size can reflect underlying genomic differentiation. Estimates were conducted for the following species of *Homoranthus*: *H. bebo*, *H. binghiensis*, *H. croftianus*, *H. flavescens*, *H. floydii*, *H. lunatus*, *H. melanostictus*, *H. montanus*, *H. papillatus*, *H. porteri* and *H. prolixus*. All samples were sourced from living collections maintained at the Botanic Gardens Sydney, Mount Annan, and Mount Tomah. Because fresh tissue was required, data could not be obtained for all target species, and replicate samples were not available. Full methodological details and results are provided in the Appendix. Briefly, sample preparation followed the CyStain PI Absolute P protocol (Sysmex Partec GmbH, Görlitz, Germany) according to the manufacturer’s instructions, and samples were analysed on a BD Accuri C6 Plus with BD CSampler Plus, using the FL2 585/40 nm detector.

## RESULTS

**Fig. 3.**
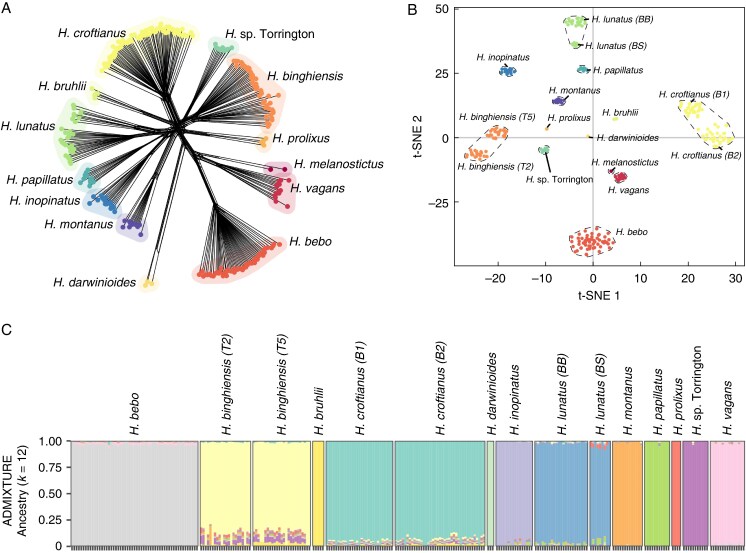
Population differentiation of species of *Homoranthus*. (A) phylogenetic network based on pairwise Euclidean genetic distances between individuals. (B) t-SNE plot of individuals, with dotted lines encircling species groups. Distinct clusters are labelled by species, with collection sites in brackets where differentiation is evident. (C) ADMIXTURE ancestry estimation for optimal *k* = 12, grouped by species. *Homoranthus melanostictus* is excluded owing to small sample size (*n* = 2).

**Fig. 4.**
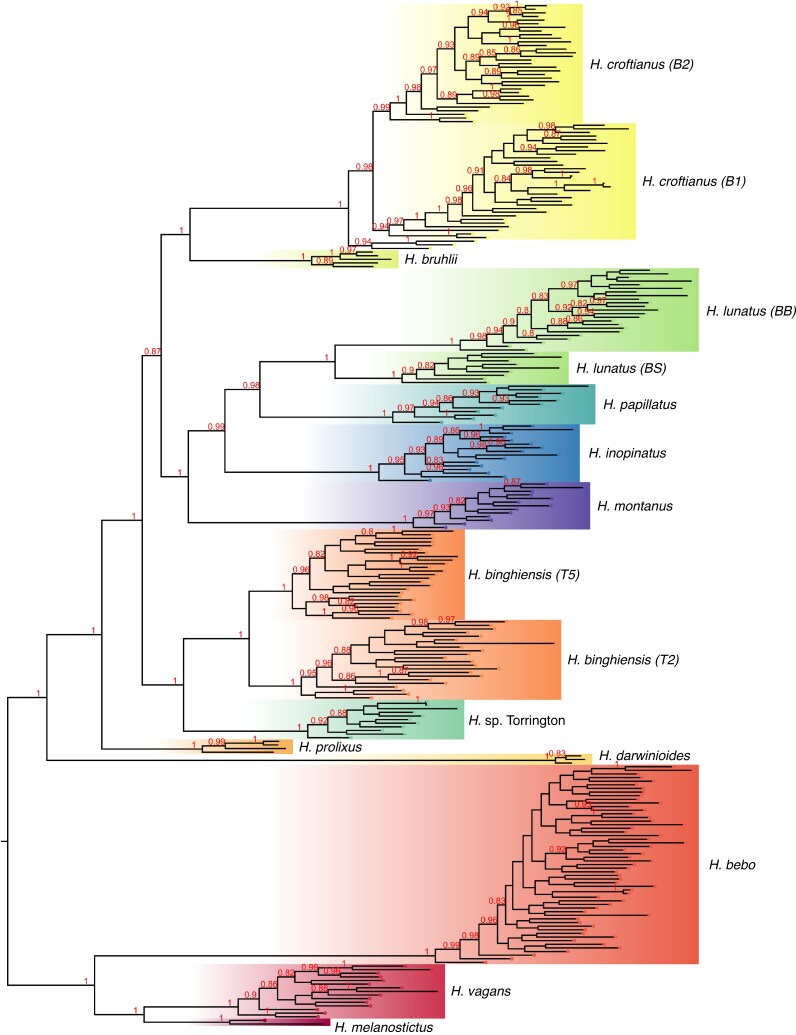
RAxML maximum likelihood tree of *Homoranthus*. Red values indicate transfer bootstrap expectations >0.8. The tree is midpoint-rooted, with species groups labelled. For species exhibiting intraspecific population differentiation (*H. croftianus*, *H. lunatus*, and *H. binghiensis*), collection sites are shown in brackets where population distinctions are evident.

Genetic analyses consistently supported the distinctiveness of the study-group species of *Homoranthus* across multiple methods, including phylogenetic network, t-SNE clustering, ADMIXTURE analysis (Fig. 3) and RAxML phylogenetic reconstruction (Fig. 4). Despite their close geographical proximity, typically within tens of kilometres (Figs 1 and 4), all species exhibited strong genetic separation. Cross-validation error and ADMIXTURE ancestry estimation for *k* = 9–15 can be found in the Supplementary Data (Figs S1 and S2). The RAxML tree revealed well-supported clades corresponding to most morphologically recognized taxa, with bootstrap support values often exceeding 0.9 (Fig. 4). Species that were geographically close were typically reciprocally monophyletic (Fig. 4). Several species, such as *H. croftianus*, *H. lunatus*, and *H. binghiensis*, consisted of distinct subclades corresponding to subpopulations (Fig. 4), consistent with population structure observed in other analyses (Fig. 3).

A disjunct population at Torrington, previously considered part of *H. lunatus*, exhibits distinct morphological and genetic characteristics. Here, we refer to this population as *Homoranthus* sp. Torrington pending formal taxonomic description in a forthcoming manuscript. This newly identified lineage is a sister taxon to *H. binghiensis* (Fig. 4), which occurs 15 km away within the same reserve (Fig. 1).

**Fig. 5.**
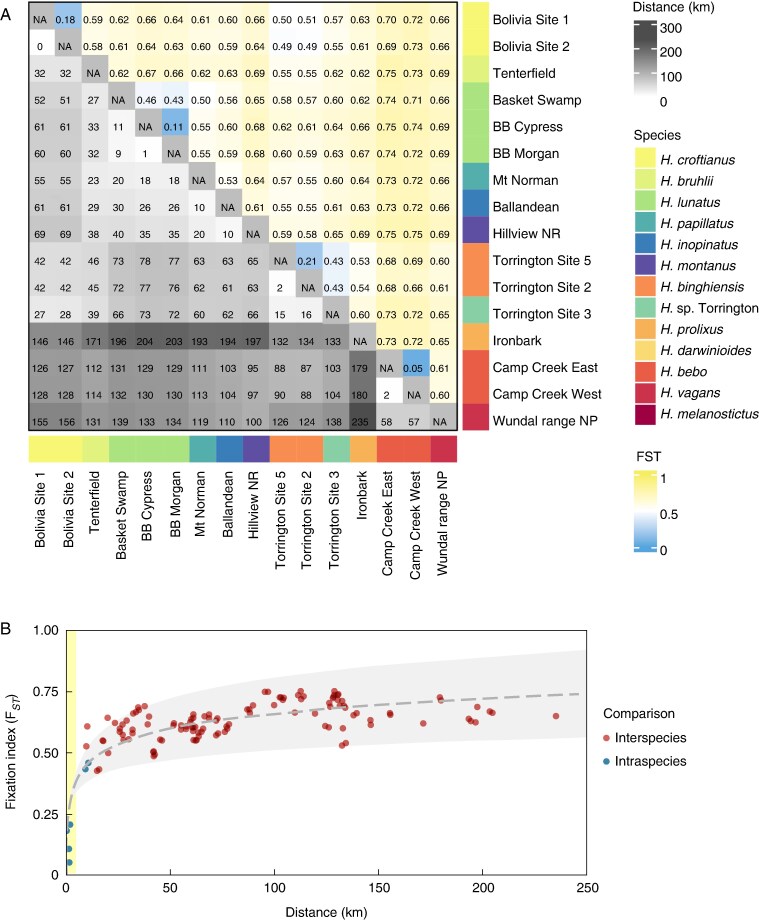
Pairwise *F*_ST_ between *Homoranthus* collection sites. (A) Weir and Hill (2002)  *F*_ST_ values (upper triangle) and geographical distances in kilometres (lower triangle) between collection sites, with coloured annotations indicating species. (B) Pairwise *F*_ST_ vs. geographical distance (in kilometres), with points coloured based on whether the comparison is within the same species (intraspecies; blue) or between different species (interspecies; red). The blue range indicates distances of <5 km. The dotted curve represents a non-linear least-squares model, with shaded bands indicating 95 % confidence intervals computed using the variance–covariance matrix and the delta method.

Population differentiation was strong (*F*_ST_ > 0.3; Rossetto *et al.*, 2019) between populations located >5 km apart, even within the same species (Fig. 4), with no evidence of meaningful gene flow (Fig. 5). Although differentiation was lower (*F*_ST_ < 0.22) between populations <2 km apart (in *H. lunatus*, *H. croftianus*, and *H. bebo*), genetic isolation was still evident at these shorter distances. This pattern was consistent across all analytical methods (Figs 2–4). Notably, at distances of >5 km, *F*_ST_ values between populations of the same species were comparable to those observed between different species (Fig. 5B).

Clonality was rare across the dataset, with only one instance detected: two samples of *H. bebo* from Camp Creek East were identified as clones. Given the rhizomatous growth habit of the species and the close proximity of the collection coordinates, this might represent repeated sampling of the same plant. Technical replicates of *H. croftianus* at Bolivia Site 1 and *H.* sp. Torrington were correctly identified as genetically identical using the kinship method, validating the approach.

Genetic diversity estimates indicated strong and significant inbreeding within populations of each species of *Homoranthus* examined (*F*_IS_ = 0.176–0.474, *P* < 0.001; Table 2), representing substantial deviations from Hardy–Weinberg equilibrium. Hardy–Weinberg equilibrium exact tests confirmed significantly lower heterozygosity at most sites compared with the null expectation (Hardy–Weinberg equilibrium), consistent with widespread inbreeding within populations (Table 2). Nevertheless, observed heterozygosity remained moderate overall (*H*_O_ = 0.137–0.248; Table 2), indicating that despite the influence of inbreeding, a measurable level of genetic diversity is still retained within populations.

**Table 2.** Genetic diversity metrics for species of *Homoranthus* across collection sites.

Species	Site	*n*	*H*_O_	*F*_IS_	EOO	AOO	Loci
*H. bebo*	Camp Creek East	45	0.218 [0.213,0.223]	0.363 [0.353,0.373]*	1.7 [0.3]	12 [1.6]	2644
	Camp Creek West	8	0.202 [0.195,0.209]	0.285 [0.266,0.304]			
*H. binghiensis*	Torrington Site 2	22	0.132 [0.128,0.136]	0.474 [0.46,0.488]*	68 [1]	52 [7.4]	2737
	Torrington Site 5	25	0.168 [0.163,0.174]	0.351 [0.338,0.364]*			
*H. croftianus*	Bolivia Site 1	28	0.166 [0.161,0.171]	0.403 [0.39,0.417]*	0.5 [0.3]	8 [0.9]	2312
	Bolivia Site 2	39	0.18 [0.176,0.185]	0.424 [0.414,0.436]*			
*H. inopinatus*	Ballandean	16	0.232 [0.225,0.239]	0.332 [0.316,0.349]*	0.6 [0.3]	8 [0.9]	1828
*H. lunatus*	Basket Swamp	9	0.134 [0.128,0.142]	0.372 [0.348,0.396]	7.4 [0.4]	12 [1.6]	2044
	BB Cypress	9	0.189 [0.181,0.198]	0.176 [0.155,0.196]			
	BB Morgan	14	0.14 [0.134,0.147]	0.383 [0.363,0.402]*			
*H. montanus*	Hillview NR	13	0.248 [0.241,0.255]	0.288 [0.268,0.305]*	6.4 [0.4]	16 [2]	1847
*H. papillatus*	Mt Norman	11	0.213 [0.207,0.219]	0.297 [0.282,0.313]*	0.4 [0.3]	8 [0.9]	2565
*H*. sp. Torrington	Torrington Site 3	10	0.185 [0.18,0.191]	0.314 [0.297,0.33]*	0.8 [0.3]	12 [1.6]	2379
*H. vagans*	Wundal Range NP	15	0.187 [0.181,0.193]	0.468 [0.451,0.485]*	4.6 [0.4]	16 [2]	2143

Observed heterozygosity (*H*_O_) and inbreeding coefficient (*F*_IS_) are presented as means with 2.5 % and 97.5 % confidence intervals, based on 1000 bootstrap replicates across loci. Asterisks (*) indicate significant deviations from Hardy–Weinberg equilibrium (*P* < 0.001). Sample size per site (*n*) is shown. Analyses used one ramet per genet; species with fewer than ten individuals were excluded. Loci were filtered to retain those with <30 % missing data and a minor allele frequency of ≥5 % within species. Extent of occurrence (EOO) and area of occupancy (AOO) are reported in kilometres squared, along with their percentile ranks compared with other Plantae in New South Wales (Le Breton, 2019). The number of loci passing filters for each species are reported. Abbreviations: BB, Boonoo Boonoo; NP, National Park; NR, Nature Reserve; SCA, State Conservation Area. Extended results can be found in Supplementary Data Tables S1 and S2.

**Fig. 6.**
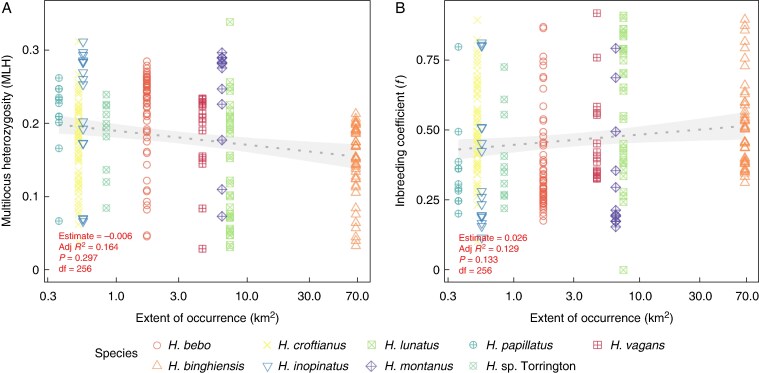
Individual diversity of *Homoranthus* individuals vs. range size of their species. (A) Individual multilocus heterozygosity (MLH) and (B) individual inbreeding coefficient (*f*) of *Homoranthus* against the species extent of occurrence (EOO, in kilometres squared). Points are coloured by species. No significant or biologically meaningful associations were found between these variables, suggesting that range size (EOO) does not explain meaningful variation in either heterozygosity or inbreeding coefficients across species.

The extent of occurrence for the ten range-restricted species of *Homoranthus* was smaller than that of 99 % of other plant species in New South Wales (Le Breton *et al.*, 2019), with all being <70 km^2^ (Table 2). AOO was also very small, with a maximum of 52 km^2^. No statistically significant association was detected between MLH and EOO (in kilometres squared) in species with ten or more samples (estimate = −0.006, adjusted *R*^2^ = 0.164, *P* = 0.297, d.f. = 256). Likewise, the relationship between *f* and EOO was not statistically significant (estimate = 0.026, adjusted *R*^2^ = 0.129, *P* = 0.133, d.f. = 256), suggesting that EOO has limited explanatory power for variation in genetic diversity or inbreeding across these species (Fig. 6).

Genome size of the assessed species of *Homoranthus* was very small overall (average size ∼295 Mbp) but varied notably among species; genome sizes varied by >30 % between *H. papillatus* (268 Mbp) and *H. melanostictus* (346 Mbp) (for full results, see the Appendix).

## DISCUSSION

We present the first population genomic assessment within *Homoranthus*, a genus of Australian flowering plants with most species known for their naturally narrow geographical ranges and high conservation concern. Our analyses of 13 species of *Homoranthus* confirmed their genetic distinctness and revealed a previously undescribed species, *Homoranthus* sp. Torrington. We found that species of *Homoranthus* generally exhibit strong genetic isolation, with limited gene flow even over short distances between populations of the same species in contiguous forest. Despite significant levels of inbreeding, heterozygosity is not exceptionally low, nor is it associated with smaller ranges. Their genome sizes are also notably small, which might contribute to their ability to colonize and persist in outcrop habitats. Altogether, these findings indicate that both biological predispositions and environmental factors have shaped the evolution within the genus *Homoranthus* and its tendency towards small geographical ranges and have specific implications for the ongoing conservation of these species.

### Why so speciose?

Geographical isolation plays a central role in shaping genetic divergence and lineage formation in *Homoranthus*, highlighting speciation as an ongoing and spatially structured process. Our analyses show that divergence occurs at multiple levels, ranging from subtle population structure within species to well-supported clades corresponding to morphologically distinct taxa. For example, populations of *H. bebo* showed no clear genetic isolation across short distances, whereas *H. binghiensis* and *H. croftianus* exhibited intermediate levels of differentiation between populations ≤2 km apart, and *H. lunatus* showed very strong genetic isolation between populations separated by <12 km. At the species level, all morphologically recognized taxa were genetically distinct, even over similarly short distances (e.g. 10 km between *H. inopinatus* and *H. papillatus*). Species occurring in close geographical proximity were frequently each other’s closest relatives in the phylogenetic reconstruction, supporting the hypothesis that spatial isolation, not ecological or morphological divergence alone, underpins speciation in the genus.

This consistent pattern of differentiation, even across distances of only a few kilometres, reflects extremely limited dispersal, which is constrained by both intrinsic biological traits and extrinsic environmental barriers. Pollinators are probably insects with short foraging ranges, fruits disperse only 1–2 m via ants, and most species are restricted to granite outcrops that act as ecological islands, creating long-term barriers to gene flow (Nathan and Muller-Landau, 2000; Hopper, 2009; Phillips, 2016; Hopper *et al.*, 2021). Although some sites have been affected historically by clearing, grazing or mining, granite outcrop populations appear largely protected, and many species show internally disjunct subpopulations even in intact habitat. This suggests that natural fragmentation and rarity pre-date European settlement, with anthropogenic impacts contributing to local reductions but not being the primary cause of isolation. Accordingly, even populations in contiguous forest may remain genetically isolated over evolutionary time scales. The same factors that promote isolation and facilitate speciation also inhibit range expansion (Hunter, 2003*a*; Givnish, 2010; Sheth *et al.*, 2020), making diversification and geographical rarity mechanistically linked outcomes of life-history traits interacting with the landscape.

Within this context of extreme isolation, both genetic drift and natural selection might drive divergence. Drift is expected to be strong owing to small population sizes and high levels of inbreeding, consistent with founder effects and restricted dispersal accelerating differentiation (Templeton, 1980; Arendt, 2015). Evidence for drift is further reflected in chromosomal and genomic variation: most species share 2*n* = 18 chromosomes, whereas *H. binghiensis* has 2*n* = 20 (Copeland *et al.*, 2008), and genome sizes vary by >30 % between the smallest and largest observed. Rapid divergence of genome architecture in *Eucalyptus*, also in the Myrtaceae family, suggests that even within relatively short evolutionary time scales, genome architecture can change drastically owing to chromosomal rearrangements, duplications, and translocations (Ferguson *et al.*, 2024). By analogy, the genome size variation in *Homoranthus* suggest that stochastic fixation of chromosomal rearrangements and other structural variants might be occurring, potentially reinforcing reproductive barriers (Ayala and Coluzzi, 2005; Barow and Jovtchev, 2007; Wood *et al.*, 2009; Rice *et al.*, 2019).

Intraspecific diversity patterns support this picture but also highlight buffering mechanisms against drift. Within species, populations exhibit high inbreeding and significant departures from Hardy–Weinberg equilibrium, consistent with expectations for small, spatially isolated populations (Frankham, 1998). Yet, observed heterozygosity and number of segregating loci is notably higher than in other small-range, threatened species analysed using comparable methods, indicating that genetic diversity is being retained despite isolation (McMaster *et al.*, 2024, 2025*a*). Similar patterns have been reported in other naturally rare or narrowly endemic species (Baskin and Baskin, 1988; Gitzendanner and Soltis, 2000; Binks *et al.*, 2015; Cieślak *et al*., 2015; Teixeira and Nazareno, 2021). In *Homoranthus*, this retention is likely to be facilitated by demographic persistence traits, such as overlapping generations, high population density, variable reproductive success, long adult lifespans and persistent soil seed banks, which can reduce relatedness among individuals and maintain effective population size across generations, consequently lowering the rate of diversity loss through drift and inbreeding (Levin, 1990; Cutrera *et al.*, 2006; Uesugi *et al.*, 2007; Wills and Read, 2007; Waples *et al.*, 2013; Riquet *et al.*, 2016). Long-lived seed banks might also act as extended periods of selection against the most inbred or homozygous seeds, because inbred seeds often exhibit reduced germination success and early survival (Angeloni et al., 2011), resulting in the preferential persistence and recruitment of more heterozygous genotypes and contributing to the higher-than-expected heterozygosity observed (Honnay et al., 2008).

Interestingly, we found no relationship between EOO and either inbreeding (*f*) or heterozygosity (MLH), implying that the present-day range is a poor predictor of genetic diversity. Notably, the range of EOO values examined in this study is relatively small, with none exceeding 70 km^2^, which might limit the ability to detect such relationships found over larger-scale comparisons (Meeus *et al.*, 2025). Nonetheless, this result highlights that variation in within-species diversity in these naturally rare species is likely to reflect differences in historical isolation, disturbance regime and persistence traits, rather than present spatial extent.

Although genetic drift explains much of the genomic divergence among species of *Homoranthus*, pronounced morphological and ecological differentiation among species suggests a substantial role for selection. *Homoranthus papillatus*, for example, is genetically and geographically close to *H. inopinatus* and *H. montanus*, yet it exhibits a markedly different ascending and layered form. It occurs on the southern slopes of Mt Norman, occupying rock crevices and the edges of gently sloping rock sheets, where soils are shallow and vegetation is low (Byrnes, 1981). The ascending, spreading habit is likely to maximize light capture across the rock surfaces (Baskin and Baskin, 1988), reflecting adaptation to its shallow-soil, outcrop-to-forest ecocline habitat. In contrast, *H. inopinatus* and *H. montanus* occupy steeply declining rock sheet aprons with deeper soils and taller, tree-dominated vegetation (Craven and Jones, 1991; Copeland *et al*., 2011) (pers. obs. P. Pemberton 2023). These species are erect and resprouting, with larger root systems probably supporting both their height and investment in fire resilience, an adaptation to higher fuel loads in their ecocline habitats.

A third growth form is observed in *H. vagans*, which inhabits open grassy woodlands. Its decumbent, mat-forming habit allows light capture under an open canopy, facilitates vegetative spread through branch-node rooting and provides protection from fire (Copeland *et al.*, 2011). Following a cool wildfire in 2023 (D. Rielly, personal communication, 3 September 2025), mats of *H. vagans* remained largely unburnt, illustrating the advantage of low, spreading growth in fire-prone woodlands (pers. obs. P. Pemberton 2024). Although outcrop species rely on boulders and rock sheets for protection, selection appears to favour the decumbent form in open habitats, where fire and competition with taller vegetation are primary pressures.

Pollinator-mediated selection might also contribute to divergence. Although most species in the study area appear insect-pollinated, observations of vertebrate visitors (a small bird, *Zosterops lateralis*, and a nocturnal mammal) on *H. inopinatus* (pers. obs. P. Pemberton 2025) and notable differences in floral fragrance suggest that there have been shifts in pollinator strategy. Moreover, apparent convergent evolution between morphologically similar but non-sister species, such as *H. lunatus* and *H. sp. Torrington*, reflects selection for comparable growth forms and ecological niches (shallow soils, low shrub competition) despite independent evolutionary histories.

These examples illustrate how natural selection might act upon multiple ecological and reproductive axes (including fire response, soil specialization, growth habit, and pollination) to shape phenotypic divergence. These selective pressures are likely to operate alongside genetic drift, which is amplified by small, isolated populations, jointly driving speciation and diversification in *Homoranthus*. Notably, selection need not be associated with ecological divergence alone; even genetically identical but isolated populations adapting to the same environment will acquire and fix different beneficial mutations, leading to divergence despite shared ancestry and ecological optimum (also known as ‘mutation-order speciation’; Mani and Clarke, 1990; Nosil and Flaxman, 2010; Ralph and Coop, 2010). These processes, in combination with drift, help to explain cases of strong genetic differentiation without accompanying morphological divergence, as observed in *H. croftianus*, *H. binghiensis*, and *H. lunatus*.

Altogether, our results suggest that drift and selection jointly drive diversification in *Homoranthus*. Drift, amplified by isolation and small effective sizes, promotes divergence and facilitates the fixation of chromosomal changes. Selection shapes phenotypic differentiation through adaptation to local ecological and reproductive pressures. Their interaction provides a compelling explanation for both the intensity of speciation and the fine-scale endemism observed in the genus.

### Surprisingly small genomes

Genome size in *Homoranthus* is strikingly small relative to the broader angiosperm context; the Plant DNA C-values database (Release 7.0) indicates that the median 2C genome size across angiosperms is an order of magnitude larger than that measured in *Homoranthus* (3326 vs. 295 Mbp; Pellicer *et al.*, 2018). This raises questions about the ecological and evolutionary significance of genome size in this genus.

Comparative analyses suggest that genome size can influence extinction risk. A recent study of ∼3250 angiosperm species found a positive correlation between larger genome size and higher extinction risk, even after controlling for life form, endemism and climatic variables (Soto Gomez *et al.*, 2024). Larger genomes might increase cell size and slow cell cycles, constraining life-history flexibility and reducing resilience to disturbance, whereas small genomes might facilitate persistence in ephemeral or heterogeneous microhabitats (Knight *et al.*, 2005).

Mechanistically, nucleotypic effects link genome size to cell and organismal traits that favour rapid development and flexible life histories. Small genomes are associated with smaller nuclei and cells, shorter cell cycles and higher cell-packing densities, translating into faster seedling growth, shorter generation times, reduced phosphorus requirement and traits characteristic of disturbance-tolerant strategies (Münzbergová, 2009; Simonin and Roddy, 2018; Faizullah *et al.*, 2021; Suriyagoda *et al.*, 2023; Givnish, 2024), supporting their potential to enable rapid exploitation of transient or micro-site habitats (Pyšek *et al.*, 2023; Guo *et al.*, 2024), such as post-fire microsites or shallow soils on granite outcrops. Population-genetic theory, however, predicts the opposite trend. Small effective population sizes, typical of isolated or inbred populations, relax selection against slightly deleterious DNA insertions, which should result in genome enlargement over time (Charlesworth and Barton, 2004; Whitney *et al.*, 2010).

This combination of forces creates an apparent paradox for *Homoranthus*: populations are small and isolated, theoretically favouring genome enlargement through drift, yet the genus consistently exhibits compact genomes. Strong selection for compact genomes in granite-outcrop niches, repeated bottlenecks or recent demographic declines might all contribute (Michael, 2014). Nevertheless, persistence remains shaped primarily by demographic and landscape factors, and understanding this paradox will require deeper investigation of the whole genomes of these species.

### Conservation implications

The fine-scale endemism in *Homoranthus* is likely to represent only a fraction of historical diversification events. In systems marked by limited dispersal and patchy habitats, population isolation and divergence are common, but not all such events result in long-term persistence; many nascent populations might fail to establish, go extinct or become genetically assimilated (Niebuhr *et al.*, 2015; Ciccheto *et al.*, 2024). One such case might already have occurred: *Homoranthus elusus*, previously recorded in the study area but not seen for >25 years, is now presumed extinct (Copeland *et al.*, 2011; Le Breton, 2019). The species that remain are likely to represent lineages that persisted despite the combined pressures of genetic drift, natural selection, and isolation. This underscores that diversification in *Homoranthus* is not only ongoing but also tightly linked to extinction risk, with speciation and loss unfolding under the same ecological and evolutionary constraints. These dynamics raise a central conservation dilemma: should the priority be to safeguard natural evolutionary processes or to intervene directly to prevent species loss?

From a minimally interventionist perspective, the emphasis is on maintaining habitat integrity to allow natural evolutionary processes of speciation and extinction to continue. The high diversification and high extinction risk seen in *Homoranthus* appear to be inherent features of their evolutionary history, leading to the many narrow endemic species present today. In this view, the long-term evolutionary process might be valued over the survival of individual species, and conservation efforts should focus on reducing anthropogenic pressures (e.g. habitat destruction, climate change) to conserve the environmental conditions necessary for these processes to persist.

However, the current threats to *Homoranthus*, driven largely by human activities, are far more intense and rapid than those encountered during their evolutionary past (Fugère and Hendry, 2018; Keck *et al.*, 2025). Climate change, invasive species and altered fire regimes introduce selective pressures that exceed natural baselines. In this context, relying solely on passive preservation might not be sufficient, and species-specific, proactive interventions might be necessary to prevent irreversible losses.

The genomic findings presented here indicate that *Homoranthus* species experience pronounced limitation of gene flow across even short distances. This poor connectivity indicates that further habitat fragmentation or disturbance could result in disproportionate genetic losses owing to increased inbreeding, drift and diversity loss. This is particularly concerning because each isolated population harbours unique and irreplaceable genetic diversity. Although moderate levels of heterozygosity have been retained across species, widespread genomic evidence of inbreeding suggests that genetic erosion is ongoing. The long generation time and lifespan of adult plants are likely to slow the rate of genetic erosion, buffering populations against rapid diversity loss. The maintenance of moderate levels of heterozygosity suggests that purging of deleterious alleles is unlikely to be complete, and inbreeding depression remains a plausible risk, with potential consequences for fitness and long-term viability (Dudash and Carr, 1998; Byers and Waller, 1999; Charlesworth and Willis, 2009). In this context, populations might be especially vulnerable to stochastic environmental change.

Conservation strategies must therefore explicitly account for the genetic structure and demographic sensitivity of these species. Any disruption of key ecological processes, such as pollinator availability, seed bank persistence or recruitment dynamics, will have long-lasting genetic consequences (Hooftman *et al.*, 2016; Hulting *et al.*, 2025). Fire regimes, in particular, must be managed carefully; short fire intervals can deplete seed banks, interrupt recruitment cycles and decouple plants from their mutualists, with disproportionately severe impacts in genetically depauperate and dispersal-limited systems (Enright *et al.*, 2015: p. 201; Duivenvoorden *et al.*, 2024). This is especially critical for obligate seeders on rocky outcrops, such as *Homoranthus*, whose reproductive success and population persistence depend on the timing and frequency of fire (Hunter, 2003b; Shi *et al.*, 2022).

In highly structured and genetically isolated systems, such as seen here in *Homoranthus*, facilitated gene flow between disjunct populations represents a possible proactive conservation intervention. Assisted migration or managed crossing between genetically differentiated populations of the same or closely related species can increase genetic diversity and fitness (Frankham, 2015, 2016; Frankham *et al.*, 2017). However, such measures also carry risks, including the potential to disrupt ongoing speciation or locally adapted traits (Bell *et al.*, 2019). Even when outbreeding depression occurs in early generations, it often diminishes in later generations (Erickson and Fenster, 2006), ultimately allowing gene flow to enhance long-term diversity and resilience. Any intervention should therefore be undertaken cautiously, guided by genomic compatibility, ecological suitability and a clear understanding of potential trade-offs. When carefully applied, these approaches could still provide a crucial tool for increasing resilience.

### Conclusion

This study highlights how the interplay between spatial isolation and life-history traits can shape both the evolutionary resilience and vulnerability of plant lineages. In *Homoranthus*, the same ecological and genetic factors that drive speciation, such as limited dispersal and strong habitat specificity, also increase the risk of extinction. These patterns show that narrow endemism does not always arise from rare or exceptional events. Instead, it can be the predictable result of persistent, repeatable processes that generate and maintain range-restricted species over time. Such processes help to explain why many regions contain multiple, unrelated narrow endemic species shaped by the same underlying dynamics.

More broadly, our findings reinforce the value of integrating population genomics into conservation practice and taxonomy, and vice versa. In fragmented and highly structured systems, conserving evolutionary resilience [the capacity of populations both to persist in their current state and to adapt through evolutionary change in response to environmental perturbations (Sgrò et al., 2011)] requires strategies that maintain connectivity, accommodate ecological processes and recognize genetic distinctiveness at fine spatial scales. As biodiversity conservation moves towards proactive and process-based frameworks, *Homoranthus* offers a compelling case for prioritizing both persistence and the conditions that enable diversification.

## Supplementary Material

mcaf316_Supplementary_Data

## Data Availability

Genomic data, metadata, and analysis scripts are available as Supplementary Data.

## SUPPLEMENTARY DATA

Table S1: comprehensive genetic diversity statistics for *Homoranthus* species across collection sites. Table S2: extent of occurrence (EOO) and area of occupancy (AOO) in kilometres squared, and their percentile ranks among NSW plant species (Le Breton *et al.*, 2019) for the study species of *Homoranthus*. Figure S2: ADMIXTURE ancestry estimation from unsupervised analysis from *K* = 9 to *K* = 15. Species groups have been subset to have a maximum of ten samples to reduce bias from uneven sample sizes. Figure S1: cross-validation error from unsupervised ADMIXTURE analysis. The minimum CV error value is *K* = 12, indicating that 12 ancestral populations are optimal.

## APPENDIX

### Flow cytometry estimation of genome size

Leaf samples of target species were collected living collections from two locations: Mount Annan Botanic Gardens (collected 19 August 2024) and Mount Tomah Botanic Gardens (collected 27 August 2024). Samples were stored in sealed plastic bags in a refrigerator and analysed on 20 and 28 August 2024, respectively.

Sample preparation followed the protocol for CyStain PI Absolute P (Sysmex Partec GmbH, Görlitz, Germany), according to the manufacturer’s instructions. A section of ∼1 cm^2^ of leaf tissue from both the sample and the internal standard was placed in 300 μL of extraction buffer in a Petri dish and chopped manually with a razor blade. The resulting suspension was filtered through a 40 μm cell strainer into a sample tube, mixed with 2000 μL of staining solution (staining buffer, propidium iodide and RNAse) and incubated at room temperature for 1 h. Samples were loaded into a flow cytometer (BD Accuri C6 Plus and BD CSampler Plus; BD Biosciences, San Jose, CA, USA). Flow cytometry was performed at ‘medium’ flow rate (35 µL/min). Data were collected via the FL2 585/40 nm detector.

Analyses were conducted using BD Accuri C6 Software v.1.0.23.1 at the Research Centre for Ecosystem Resilience, Sydney Botanic Gardens. *Zea mays* L. ‘CE-777’ (2C = 5.72 pg) was used as the internal standard in all samples, and *Glycine max* (L.) Merr. ‘Polanka’ (2C = 2.50 pg) was included as a positive control in the 20 August 2024 run.

Nucleus masses were estimated by dividing the target peak by the standard peak mean PE-A, then multiplying by the standard known mass (in picograms). To estimate genome size in megabases (Mbp), the mass was multiplied by 978 (Doležel *et al.*, 2003; Doležel and Bartoš, 2005).

**Table A1.** Results of flow cytometry analysis for genome size estimation for *Homoranthus* species.

Date	Target species	Peak ID	Count^1^	Mean PE-A^2^	CV PE-A^5^	Nucleus (2C pg) ^3^	Genome size (2C Mbp)^4^
28 August 2024	*Homoranthus croftianus* J.T.Hunter (Mt Tomah)	Target	1690	8282	0.1045	0.631	618
		Maize 2C	697	75 027	0.0898		
		Maize 4C	1101	152 117	0.0778		
28 August 2024	*Homoranthus porteri* (C.T.White) Craven & S.R.Jones (Mt Tomah)	Target	874	8370	0.1077	0.554	542
		Maize 2C	160	86 442	0.0656		
		Maize 4C	539	158 674	0.0849		
28 August 2024	*Homoranthus papillatus* Byrnes (Mt Tomah)	Target	1198	8306	0.1010	0.577	564
		Maize 2C	1286	82 329	0.0805		
		Maize 4C	1685	163 251	0.0863		
28 August 2024	*Homoranthus prolixus* Craven & S.R.Jones (Mt Tomah)	Target	602	8639	0.0981	0.609	595
		Maize 2C	425	81 159	0.0789		
		Maize 4C	717	165 800	0.0800		
20 August 2024	*Homoranthus flavescens* A.Cunn. ex Schauer (Mt Annan [bed 14c])	Target	3294	26 120	0.0799	0.705	690
		Maize 2C	410	211 801	0.0632		
		Maize 4C	609	431 687	0.0493		
20 August 2024	*Homoranthus floydii* Craven & S.R.Jones (Mt Annan [bed 10])	Target	5279	22 276	0.0771	0.579	567
		Maize 2C	444	219 913	0.0698		
		Maize 4C	798	446 199	0.0554		
20 August 2024	*Homoranthus bebo* L.M.Copel. (Mt Annan [bed 19])	Target	5809	25 971	0.0761	0.644	630
		Maize 2C	487	230 683	0.0431		
		Maize 4C	1168	465 419	0.0588		
20 August 2024	*Homoranthus melanostictus* Craven & S.R.Jones (Mt Annan [bed 14c])	Target	4206	26 115	0.0856	0.746	729
		Maize 2C	649	200 281	0.0611		
		Maize 4C	1130	405 860	0.0689		
20 August 2024	*Homoranthus binghiensis J.T.Hunter* (Mt Annan [bed 217c])	Target	1945	31 660	0.0568	0.699	683
		Maize 2C	194	259 194	0.0532		
		Maize 4C	485	517 175	0.0508		
20 August 2024	*Homoranthus lunatus* Craven & S.R.Jones (Mt Annan [bed 14c])	Target	1793	24 323	0.0502	0.623	609
		Maize 2C	186	223 329	0.0413		
		Maize 4C	678	447 576	0.0737		
20 August 2024	*Homoranthus prolixus* Craven & S.R.Jones (Mt Annan [bed 14c])	Target	2043	26 120	0.0770	0.623	609
		Maize 2C	202	239 916	0.0795		
		Maize 4C	584	480 614	0.0806		
20 August 2024	*Homoranthus montanus* Craven & S.R.Jones (Mt Annan [bed 14c])	Target	1782	29 092	0.0597	0.639	625
		Maize 2C	400	260 305	0.0824		
		Maize 4C	587	508 487	0.0745		

^1^Count of nucleus observations.

^2^Mean flourescence intensity.

^3^Estimated genome size ((Target PE-A / Standard PE-A) * Standard nucleus size [pg]).

^4^Estimated nucleus size [pg] * 978 [Mbp/pg].

^5^Coefficient of variation of flourescence intensity.

## References

[mcaf316-B1] Alexander DH, Lange K. 2011. Enhancements to the ADMIXTURE algorithm for individual ancestry estimation. BMC Bioinformatics 12: 246. doi:10.1186/1471-2105-12-246 21682921 PMC3146885

[mcaf316-B2] Angeloni F, Ouborg NJ, Leimu R. 2011. Meta-analysis on the association of population size and life history with inbreeding depression in plants. Biological Conservation 144: 35–43. 10.1016/j.biocon.2010.08.016

[mcaf316-B3] Arendt JD . 2015. Effects of dispersal plasticity on population divergence and speciation. Heredity 115: 306–311. doi:10.1038/hdy.2015.21 25806544 PMC4815459

[mcaf316-B4] Arnold JB . 2024. Ggthemes: extra themes, scales and geoms for ‘ggplot2’. https://github.com/jrnold/ggthemes

[mcaf316-B5] Auld TD, Ooi MKJ. 2009. Heat increases germination of water-permeable seeds of obligate-seeding *Darwinia* species (Myrtaceae). Plant Ecology 200: 117–127. doi:10.1007/s11258-008-9437-7

[mcaf316-B6] Ayala FJ, Coluzzi M. 2005. Chromosome speciation: humans, *Drosophila*, and mosquitoes. Proceedings of the National Academy of Sciences of the United States of America 102: 6535–6542. doi:10.1073/pnas.0501847102 15851677 PMC1131864

[mcaf316-B7] Barow M, Jovtchev G. 2007. Endopolyploidy in plants and its analysis by flow cytometry. In: Doležel J, Greilhuber J, Suda J. eds. Flow cytometry with plant cells. John Wiley & Sons, Ltd, 349–372. https://onlinelibrary.wiley.com/doi/abs/10.1002/9783527610921.ch15

[mcaf316-B8] Barrett T, Dowle M, Srinivasan A, et al 2025. Data.table: rxtension of ‘data.frame’. https://cran.r-project.org/web/packages/data.table/index.html

[mcaf316-B9] Baskin JM, Baskin CC. 1988. Endemism in rock outcrop plant communities of unglaciated eastern United States: an evaluation of the roles of the edaphic, genetic and light factors. Journal of Biogeography 15: 829–840. doi:10.2307/2845343

[mcaf316-B10] Bean AR . 2000. *Homoranthus coracinus* (Myrtaceae), a new species from Queensland. Austrobaileya 5: 687–689. doi:10.5962/p.299638

[mcaf316-B11] Bean AR . 2009. *Homoranthus tricolor* (Myrtaceae), a new species from south-eastern Queensland. Austrobaileya 8: 77–79. doi:10.5962/p.299782

[mcaf316-B12] Bell DA, Robinson ZL, Funk WC, et al 2019. The exciting potential and remaining uncertainties of genetic rescue. Trends in Ecology & Evolution 34: 1070–1079. doi:10.1016/j.tree.2019.06.006 31296345

[mcaf316-B13] Binks RM, Millar MA, Byrne M. 2015. Not all rare species are the same: contrasting patterns of genetic diversity and population structure in two narrow-range endemic sedges. Biological Journal of the Linnean Society 114: 873–886. doi:10.1111/bij.12465

[mcaf316-B14] Breton Le, Tdl. 2016. Conservation Assessment of Homoranthus bruhlii. Science Division, NSW Department of Planning, Industry and Environment. https://www.environment.nsw.gov.au/-/media/OEH/Corporate-Site/Documents/Animals-and-plants/Scientific-Committee/Determinations/2020/homeranthus-bruhlii-cam-critically-endangered.pdf

[mcaf316-B15] Briggs JD, Leigh JH. 1996. Rare or threatened Australian plants, 1995 revised edn. Collingwood, VIC: CSIRO Publishing.

[mcaf316-B16] Brooks TM, Pimm SL, Akçakaya HR, et al 2019. Measuring terrestrial area of habitat (AOH) and its utility for the IUCN red list. Trends in Ecology & Evolution 34: 977–986. doi:10.1016/j.tree.2019.06.009 31324345

[mcaf316-B17] Byers DL, Waller DM. 1999. Do plant populations purge their genetic load? Effects of population size and mating history on inbreeding depression. Annual Review of Ecology and Systematics 30: 479–513. doi:10.1146/annurev.ecolsys.30.1.479

[mcaf316-B18] Byrnes NB . 1981. Notes on the Genus Homoranthus (myrtaceae) in Australia. Austrobaileya 1: 372–375. https://www.jstor.org/stable/41738621

[mcaf316-B19] Caballero A, Bravo I, Wang J. 2017. Inbreeding load and purging: implications for the short-term survival and the conservation management of small populations. Heredity 118: 177–185. doi:10.1038/hdy.2016.80 27624114 PMC5234482

[mcaf316-B20] Cardoso P . 2017. *red* - an R package to facilitate species red list assessments according to the IUCN criteria. Biodiversity Data Journal 5: e20530. doi:10.3897/BDJ.5.e20530 PMC5665006 29104439

[mcaf316-B21] Chang CC, Chow CC, Tellier LC, Vattikuti S, Purcell SM, Lee JJ. 2015. Second-generation PLINK: rising to the challenge of larger and richer datasets. GigaScience 4: s13742-015-0047-8. doi:10.1186/s13742-015-0047-8 PMC4342193 25722852

[mcaf316-B22] Charlesworth B, Barton N. 2004. Genome size: does bigger mean worse? Current Biology 14: R233–R235. doi:10.1016/j.cub.2004.02.054 15043833

[mcaf316-B23] Charlesworth D, Willis JH. 2009. The genetics of inbreeding depression. Nature Reviews. Genetics 10: 783–796. doi:10.1038/nrg2664 19834483

[mcaf316-B24] Ciccheto JRM, Carnaval AC, Araujo SBL. 2024. The influence of fragmented landscapes on speciation. Journal of Evolutionary Biology 37: 1499–1509. doi:10.1093/jeb/voae043 38567816

[mcaf316-B25] Cieślak E, Cieślak J, Szeląg Z, Ronikier M. 2015. Genetic structure of *Galium cracoviense* (Rubiaceae): a naturally rare species with an extremely small distribution range. Conservation Genetics 16: 929–938. doi:10.1007/s10592-015-0711-7

[mcaf316-B26] Copeland LM, Bruhl JJ, Craven LA, Brubaker CL. 2008. New chromosome numbers in *Homoranthus* (Myrtaceae: Chamelaucieae) and notes on their taxonomic utility. Australian Systematic Botany 21: 443–447. doi:10.1071/SB08036

[mcaf316-B27] Copeland LM, Craven LA, Bruhl JJ. 2011. A taxonomic review of *Homoranthus* (Myrtaceae: Chamelaucieae). Australian Systematic Botany 24: 351–374. doi:10.1071/SB11015

[mcaf316-B28] Craven LA, Jones SR. 1991. A taxonomic review of *Homoranthus* and two new species of *Darwinia* (both Myrtaceae, Chamelaucieae). Australian Systematic Botany 4: 513–533. doi:10.1071/sb9910513

[mcaf316-B29] Cutrera AP, Lacey EA, Busch C. 2006. Intraspecific variation in effective population size in Talar Tuco-Tucos (*Ctenomys talarum*): the role of demography. Journal of Mammalogy 87: 108–116. doi:10.1644/05-MAMM-A-075R1.1

[mcaf316-B30] Dellinger AS . 2020. Pollination syndromes in the 21^st^ century: where do we stand and where may we go? New Phytologist 228: 1193–1213. 10.1111/nph.16793 33460152

[mcaf316-B150] Department of Climate Change, Energy, the Environment and Water, Australian Government . 2008a. Approved Conservation Advice for Homoranthus darwinioides. Department of Climate Change, Energy, the Environment and Water, Australian Government. http://www.environment.gov.au/biodiversity/threatened/species/pubs/12974-conservation-advice.pdf.

[mcaf316-B151] Department of Climate Change, Energy, the Environment and Water, Australian Government . 2008b. Approved Conservation Advice for Homoranthus lunatus. Department of Climate Change, Energy, the Environment and Water, Australian Government. https://www.environment.gov.au/biodiversity/threatened/species/pubs/55189-conservation-advice.pdf.

[mcaf316-B152] Department of Climate Change, Energy, the Environment and Water, Australian Government . 2008c. Approved Conservation Advice for Homoranthus montanus. Department of Climate Change, Energy, the Environment and Water, Australian Government. http://www.environment.gov.au/biodiversity/threatened/species/pubs/24319-conservation-advice.pdf.

[mcaf316-B153] Department of Climate Change, Energy, the Environment and Water, Australian Government . 2008d. Approved Conservation Advice for Homoranthus prolixus. Department of Climate Change, Energy, the Environment and Water, Australian Government. http://www.environment.gov.au/biodiversity/threatened/species/pubs/55198-conservation-advice.pdf.

[mcaf316-B31] Department of Climate Change, Energy, the Environment and Water. 2018. Approved Conservation Advice for Homoranthus bebo. Australian Government. https://www.environment.gov.au/biodiversity/threatened/species/pubs/88498-conservation-advice-04072019.pdf

[mcaf316-B32] Department of Climate Change, Energy, the Environment & Water. 2012. Interim biogeographic regionalisation for Australia (IBRA) version 7.1 (regions). Australian Government. https://fed.dcceew.gov.au/datasets/erin::interim-biogeographic-regionalisation-for-australia-ibra-version-7-1-subregions/about

[mcaf316-B33] Doležel J, Bartoš J. 2005. Plant DNA flow cytometry and estimation of nuclear genome size. Annals of Botany 95: 99–110. doi:10.1093/aob/mci005 15596459 PMC4246710

[mcaf316-B34] Doležel J, Bartoš J, Voglmayr H, Greilhuber J. 2003. Letter to the editor. Cytometry Part A 51A: 127–128. doi:10.1002/cyto.a.10013 12541287

[mcaf316-B35] Dudash MR, Carr DE. 1998. Genetics underlying inbreeding depression in *Mimulus* with contrasting mating systems. Nature 393: 682–684. doi:10.1038/31468

[mcaf316-B36] Duivenvoorden E, Wagner B, Nitschke CR, Kasel S. 2024. Short-interval, high-severity wildfires cause declines in soil seed bank diversity in montane forests of south-eastern Australia. Forest Ecology and Management 553: 121627. doi:10.1016/j.foreco.2023.121627

[mcaf316-B37] Ellstrand N, Elam D. 1993. Population genetic consequences of small population size: implications for plant conservation. Annual Review of Ecology and Systematics 24: 217–242. doi:10.1146/annurev.es.24.110193.001245

[mcaf316-B38] Enright NJ, Fontaine JB, Bowman DM, Bradstock RA, Williams RJ. 2015. Interval squeeze: altered fire regimes and demographic responses interact to threaten woody species persistence as climate changes. Frontiers in Ecology and the Environment 13: 265–272. doi:10.1890/140231

[mcaf316-B39] Erickson DL, Fenster CB. 2006. Intraspecific hybridization and the recovery of fitness in the native legume chamaecrista fasciculata. Evolution 60: 225–233. doi:10.1111/j.0014-3820.2006.tb01101.x 16610315

[mcaf316-B40] Erickson E, Junker RR, Ali JG, McCartney N, Patch HM, Grozinger CM. 2022. Complex floral traits shape pollinator attraction to ornamental plants. Annals of Botany 130: 561–577. doi:10.1093/aob/mcac082 35732011 PMC9510942

[mcaf316-B41] Excoffier L, Lischer HEL. 2010. Arlequin suite ver 3.5: a new series of programs to perform population genetics analyses under Linux and Windows. Molecular Ecology Resources 10: 564–567. doi:10.1111/j.1755-0998.2010.02847.x 21565059

[mcaf316-B42] Fahey PS, Udovicic F, Cantrill DJ, Nicolle D, McLay TGB, Bayly MJ. 2022. A phylogenetic investigation of the taxonomically problematic *Eucalyptus odorata* complex (*E.* section *Adnataria* series *Subbuxeales*): evidence for extensive interspecific gene flow and reticulate evolution. Australian Systematic Botany 35: 403–435. doi:10.1071/SB21029

[mcaf316-B43] Faizullah L, Morton JA, Hersch-Green EI, Walczyk AM, Leitch AR, Leitch IJ. 2021. Exploring environmental selection on genome size in angiosperms. Trends in Plant Science 26: 1039–1049. doi:10.1016/j.tplants.2021.06.001 34219022

[mcaf316-B44] Fellows I, Stotz using the Jm library by JP. 2023. OpenStreetMap: access to open street map raster images. https://cran.r-project.org/web/packages/OpenStreetMap/index.html

[mcaf316-B45] Ferguson S, Jones A, Murray K, Andrew R, Schwessinger B, Borevitz J. 2024. Plant genome evolution in the genus *Eucalyptus* is driven by structural rearrangements that promote sequence divergence. Genome Research 34: 606–619. doi:10.1101/gr.277999.123 38589251 PMC11146599

[mcaf316-B46] Ferris KG, Sexton JP, Willis JH. 2014. Speciation on a local geographic scale: the evolution of a rare rock outcrop specialist in *Mimulus*. Philosophical Transactions of the Royal Society B: Biological Sciences 369: 20140001. doi:10.1098/rstb.2014.0001 PMC4071529 24958929

[mcaf316-B47] Frankham R . 1995. Conservation genetics. Annual Review of Genetics 29: 305–327. doi:10.1146/annurev.ge.29.120195.001513 8825477

[mcaf316-B48] Frankham R . 1998. Inbreeding and extinction: island populations. Conservation Biology 12: 665–675. doi:10.1111/j.1523-1739.1998.96456.x

[mcaf316-B49] Frankham R . 2015. Genetic rescue of small inbred populations: meta-analysis reveals large and consistent benefits of gene flow. Molecular Ecology 24: 2610–2618. doi:10.1111/mec.13139 25740414

[mcaf316-B50] Frankham R . 2016. Genetic rescue benefits persist to at least the F3 generation, based on a meta-analysis. Biological Conservation 195: 33–36. doi:10.1016/j.biocon.2015.12.038

[mcaf316-B51] Frankham R, Ballou JD, Ralls K et al 2017. Genetic management of fragmented animal and plant populations. Oxford, United Kingdom: Oxford University Press.

[mcaf316-B52] Fugère V, Hendry AP. 2018. Human influences on the strength of phenotypic selection. Proceedings of the National Academy of Sciences of the United States of America 115: 10070–10075. doi:10.1073/pnas.1806013115 30224474 PMC6176558

[mcaf316-B53] Gitzendanner MA, Soltis PS. 2000. Patterns of genetic variation in rare and widespread plant congeners. American Journal of Botany 87: 783–792. doi:10.2307/2656886 10860909

[mcaf316-B54] Givnish TJ . 2010. Ecology of plant speciation. TAXON 59: 1326–1366. doi:10.1002/tax.595003

[mcaf316-B55] Givnish TJ . 2024. High metabolic rates drive tiny genomes in plants (and birds): a commentary on ‘The smallest angiosperm genomes may be the price for effective traps of bladderworts’. Annals of Botany 134: i–iv. doi:10.1093/aob/mcae163 PMC11688525 39361413

[mcaf316-B56] Goudet J . 2005. Hierfstat, a package for r to compute and test hierarchical F-statistics. Molecular Ecology Notes 5: 184–186. doi:10.1111/j.1471-8286.2004.00828.x

[mcaf316-B57] Graffelman J, Chang C, Puig X, Wigginton J, Ortoleva L, Engels WR. 2025. HardyWeinberg: statistical tests and graphics for Hardy-Weinberg equilibrium. https://cran.r-project.org/web/packages/HardyWeinberg/index.html

[mcaf316-B58] Gu Z . 2022. Complex heatmap visualization. iMeta 1: e43. doi:10.1002/imt2.43 38868715 PMC10989952

[mcaf316-B59] Guo K, Pyšek P, van Kleunen M, et al 2024. Plant invasion and naturalization are influenced by genome size, ecology and economic use globally. Nature Communications 15: 1330. doi:10.1038/s41467-024-45667-4 PMC10864296 38351066

[mcaf316-B60] Honnay O, Bossuyt B, Jacquemyn H, Shimono A, Uchiyama K. 2008. Can a seed bank maintain the genetic variation in the above ground plant population? Oikos 117: 1–5. doi:10.1111/j.2007.0030-1299.16188.x

[mcaf316-B61] Hooftman DAP, Edwards B, Bullock JM. 2016. Reductions in connectivity and habitat quality drive local extinctions in a plant diversity hotspot. Ecography 39: 583–592. doi:10.1111/ecog.01503

[mcaf316-B62] Hopper SD . 2009. OCBIL theory: towards an integrated understanding of the evolution, ecology and conservation of biodiversity on old, climatically buffered, infertile landscapes. Plant and Soil 322: 49–86. doi:10.1007/s11104-009-0068-0

[mcaf316-B63] Hopper SD, Lambers H, Silveira FAO, Fiedler PL. 2021. OCBIL theory examined: reassessing evolution, ecology and conservation in the world’s ancient, climatically buffered and infertile landscapes. Biological Journal of the Linnean Society 133: 266–296. doi:10.1093/biolinnean/blaa213

[mcaf316-B64] Hulting KA, Brudvig LA, Damschen EI, et al 2025. Habitat edges decrease plant reproductive output in fragmented landscapes. The Journal of Ecology 113: 531–541. doi:10.1111/1365-2745.14452

[mcaf316-B65] Hunter J . 1998. Two new rare species of *Homoranthus* (Myrtaceae: Chamelaucieae) from the Northern Tablelands of New South Wales. Telopea 8: 35–40. doi:10.7751/telopea19982012

[mcaf316-B66] Hunter J . 2002. Vegetation survey and flora of the Tenterfield group of Nature Reserves. 10.13140/RG.2.1.1329.0084

[mcaf316-B67] Hunter JT . 2003a. Factors affecting range size differences for plant species on rock outcrops in eastern Australia. Diversity & Distributions 9: 211–220. doi:10.1046/j.1466-822X.2003.00167.x

[mcaf316-B68] Hunter JT . 2003b. Persistence on inselbergs: the role of obligate seeders and resprouters. Journal of Biogeography 30: 497–510. doi:10.1046/j.1365-2699.2003.00876.x

[mcaf316-B70] Hunter J . 2015. Vegetation and flora of Dthinnia Dthinnawan nature reserve. doi:10.13140/RG.2.1.4540.2723

[mcaf316-B71] Hunter J . 2016. *Mapping of Homoranthus bebo L.M.Copel*. doi:10.13140/RG.2.2.26016.17922

[mcaf316-B72] Huson DH . 1998. SplitsTree: analyzing and visualizing evolutionary data. Bioinformatics 14: 68–73. doi:10.1093/bioinformatics/14.1.68 9520503

[mcaf316-B73] Huson DH, Bryant D. 2006. Application of phylogenetic networks in evolutionary studies. Molecular Biology and Evolution 23: 254–267. doi:10.1093/molbev/msj030 16221896

[mcaf316-B74] Jaccoud D, Peng K, Feinstein D, Kilian A. 2001. Diversity arrays: a solid state technology for sequence information independent genotyping. Nucleic Acids Research 29: e25. doi:10.1093/nar/29.4.e25 11160945 PMC29632

[mcaf316-B75] Kassambara A . 2023. Ggpubr: ‘ggplot2’ based publication ready plots. https://cran.r-project.org/web/packages/ggpubr/index.html

[mcaf316-B76] Keck F, Peller T, Alther R, et al 2025. The global human impact on biodiversity. Nature 641: 395–400. doi:10.1038/s41586-025-08752-2 40140566 PMC12058524

[mcaf316-B77] Keenan K, McGinnity P, Cross TF, Crozier WW, Prodöhl PA. 2013. diveRsity: an R package for the estimation and exploration of population genetics parameters and their associated errors. Methods in Ecology and Evolution 4: 782–788. doi:10.1111/2041-210X.12067

[mcaf316-B78] Kilian A, Wenzl P, Huttner E, et al 2012. Diversity arrays technology: a generic genome profiling technology on open platforms. In: Pompanon F, Bonin A. eds. Data production and analysis in population genomics: methods and protocols. Methods in Molecular Biology. Totowa, NJ: Humana Press, 67–89. 10.1007/978-1-61779-870-2_5 22665276

[mcaf316-B79] Kleinman-Ruiz D, Lucena-Perez M, Villanueva B, et al 2022. Purging of deleterious burden in the endangered Iberian lynx. Proceedings of the National Academy of Sciences of the United States of America 119: e2110614119. doi:10.1073/pnas.2110614119 35238662 PMC8931242

[mcaf316-B80] Knight CA, Molinari NA, Petrov DA. 2005. The large genome constraint hypothesis: evolution, ecology and phenotype. Annals of Botany 95: 177–190. doi:10.1093/aob/mci011 15596465 PMC4246716

[mcaf316-B81] Kozlov AM, Darriba D, Flouri T, Morel B, Stamatakis A. 2019. RAxML-NG: a fast, scalable and user-friendly tool for maximum likelihood phylogenetic inference. Bioinformatics 35: 4453–4455. doi:10.1093/bioinformatics/btz305 31070718 PMC6821337

[mcaf316-B82] Krijthe JH . 2015. Rtsne: T-distributed stochastic neighbor embedding using a Barnes-Hut implementation version 0.17 from CRAN. https://github.com/jkrijthe/Rtsne

[mcaf316-B83] Lal M, Southgate P, Jerry D, Zenger K. 2018. Genome-wide comparisons reveal evidence for a species complex in the black-lip pearl oyster *Pinctada margaritifera* (Bivalvia: Pteriidae). Scientific Reports 8: 191. doi:10.1038/s41598-017-18602-5 29317743 PMC5760631

[mcaf316-B84] Leão TCC, Fonseca CR, Peres CA, Tabarelli M. 2014. Predicting extinction risk of Brazilian Atlantic forest angiosperms. Conservation Biology: The Journal of the Society for Conservation Biology 28: 1349–1359. doi:10.1111/cobi.12286 24665927

[mcaf316-B85] Le Breton TD . 2019. Conservation assessment of Homoranthus elusus. Science Division, NSW Department of Planning, Industry and Environment. https://www.dcceew.gov.au/sites/default/files/env/consultations/4264ba54-69fc-481f-afdc-595842e69006/files/conservation-assessment-homoranthus-elusus.pdf

[mcaf316-B86] Le Breton TD, Auld TD. 2018. Conservation assessment for Homoranthus bebo. NSW Office of Environment and Heritage. https://www.environment.nsw.gov.au/sites/default/files/homoranthus-bebo-final-determination-conservation-assessment-report.pdf

[mcaf316-B87] Le Breton TD, Zimmer HC, Gallagher RV, Cox M, Allen S, Auld TD. 2019. Using IUCN criteria to perform rapid assessments of at-risk taxa. Biodiversity and Conservation 28: 863–883. doi:10.1007/s10531-019-01697-9

[mcaf316-B88] Levin DA . 1990. The seed bank as a source of genetic novelty in plants. The American Naturalist 135: 563–572. doi:10.1086/285062

[mcaf316-B89] Maaten LVD, Hinton G. 2008. Visualizing data using t-SNE. Journal of Machine Learning Research: JMLR 9: 2579–2605.

[mcaf316-B90] Mani GS, Clarke BC. 1990. Mutational order: a major stochastic process in evolution. Proceedings of the Royal Society of London B: Biological Sciences 240: 29–37. doi:10.1098/rspb.1990.0025 1972992

[mcaf316-B91] Manichaikul A, Mychaleckyj JC, Rich SS, Daly K, Sale M, Chen W-M. 2010. Robust relationship inference in genome-wide association studies. Bioinformatics 26: 2867–2873. doi:10.1093/bioinformatics/btq559 20926424 PMC3025716

[mcaf316-B92] McMaster ES . 2025. eilishmcmaster/fastDiversity: v1.0.0. Zenodo. doi:10.5281/zenodo.12741582.

[mcaf316-B93] McMaster ES, Duretto M, Yap J-YS, Rossetto M. 2025a. Conservation genomics uncovers disjunct subspecies and critically low diversity in *Zieria obcordata* A.Cunn. (Rutaceae). Australian Systematic Botany 38. doi:10.1071/SB24034

[mcaf316-B94] McMaster ES, Lu-Irving P, van der Merwe MM, Ho SYW, Rossetto M. 2025b. Evaluating kinship estimation methods for reduced-representation SNP data in non-model species. Molecular Ecology Resources 25: e70038. doi:10.1111/1755-0998.70038 40859063 PMC12550493

[mcaf316-B95] McMaster ES, Yap J-YS, Chen SH, et al 2024. On the edge: conservation genomics of the critically endangered dwarf mountain pine *Pherosphaera fitzgeraldii*. Basic and Applied Ecology 80: 61–71. doi:10.1016/j.baae.2024.09.003

[mcaf316-B96] Meeus MP, Lescroart J, Svardal H. 2025. Genomic diversity in felids correlates with range and density, not census size. Conservation Genetics 26: 873–890. doi:10.1007/s10592-025-01709-y

[mcaf316-B97] Melville J, Haines ML, Boysen K, et al 2017. Identifying hybridization and admixture using SNPs: application of the DArTseq platform in phylogeographic research on vertebrates. Royal Society Open Science 4: 161061. doi:10.1098/rsos.161061 28791133 PMC5541528

[mcaf316-B98] Michael TP . 2014. Plant genome size variation: bloating and purging DNA. Briefings in Functional Genomics 13: 308–317. doi:10.1093/bfgp/elu005 24651721

[mcaf316-B99] Miller MA, Pfeiffer W, Schwartz T. 2010. Creating the CIPRES science gateway for inference of large phylogenetic trees. In 2010 Gateway Computing Environments Workshop (GCE), November 2010. pp. 1–8.

[mcaf316-B100] Milton Bache S, Wickham H, Henry L. 2022. Magrittr: a forward-pipe operator for R. https://cran.r-project.org/web/packages/magrittr/index.html

[mcaf316-B101] Münzbergová Z . 2009. The effect of genome size on detailed species traits within closely related species of the same habitat. Botanical Journal of the Linnean Society 160: 290–298. doi:10.1111/j.1095-8339.2009.00973.x

[mcaf316-B102] Nathan R, Muller-Landau HC. 2000. Spatial patterns of seed dispersal, their determinants and consequences for recruitment. Trends in Ecology & Evolution 15: 278–285. doi:10.1016/S0169-5347(00)01874-7 10856948

[mcaf316-B103] Neuwirth E . 2022. RColorBrewer: ColorBrewer palettes. https://cran.r-project.org/web/packages/RColorBrewer/index.html

[mcaf316-B104] Newell RJP, McMaster ES. 2025. Fast-NNT: fast NeighbourNet split trees for unrooted phylogenetic analysis. Zenodo. doi:10.5281/zenodo.16923306.

[mcaf316-B105] Niebuhr BBS, Wosniack ME, Santos MC, et al 2015. Survival in patchy landscapes: the interplay between dispersal, habitat loss and fragmentation. Scientific Reports 5: 11898. doi:10.1038/srep11898 26148488 PMC4493700

[mcaf316-B106] Nosil P, Flaxman SM. 2010. Conditions for mutation-order speciation. Proceedings of the Royal Society B: Biological Sciences 278: 399–407. doi:10.1098/rspb.2010.1215 PMC3013408 20702458

[mcaf316-B107] Nyberg B, Walsh SK, Rønsted N. 2025. The global conservation status of plants growing on cliffs and rocky outcrops. Basic and Applied Ecology 82: 18–27. doi:10.1016/j.baae.2024.11.002

[mcaf316-B108] Paradis E, Claude J, Strimmer K. 2004. APE: analyses of phylogenetics and evolution in R language. Bioinformatics 20: 289–290. doi:10.1093/bioinformatics/btg412 14734327

[mcaf316-B109] Pebesma E . 2018. Simple features for R: standardized support for spatial vector data. The R Journal 10: 439. doi:10.32614/RJ-2018-009

[mcaf316-B110] Pellicer J, Hidalgo O, Dodsworth S, Leitch IJ. 2018. Genome size diversity and its impact on the evolution of land plants. Genes 9: 88. doi:10.3390/genes9020088 29443885 PMC5852584

[mcaf316-B111] Phillips GP . 2016. Conservation assessment of Homoranthus croftianus. Science, Economics and Insights Division, NSW Department of Planning and Environment. https://www.environment.nsw.gov.au/research-and-publications/publications-search/conservation-action-plan-bolivia-homoranthus-homoranthus-croftianus

[mcaf316-B112] Pinheiro F, Cozzolino S, Draper D, et al 2014. Rock outcrop orchids reveal the genetic connectivity and diversity of inselbergs of northeastern Brazil. BMC Evolutionary Biology 14: 49. doi:10.1186/1471-2148-14-49 24629134 PMC4004418

[mcaf316-B113] Purcell S, Neale B, Todd-Brown K, et al 2007. PLINK: a tool set for whole-genome association and population-based linkage analyses. American Journal of Human Genetics 81: 559–575. doi:10.1086/519795 17701901 PMC1950838

[mcaf316-B114] Pyšek P, Lučanová M, Dawson W, et al 2023. Small genome size and variation in ploidy levels support the naturalization of vascular plants but constrain their invasive spread. New Phytologist 239: 2389–2403. doi:10.1111/nph.19135 37438886

[mcaf316-B115] Ralph P, Coop G. 2010. Parallel adaptation: one or many waves of advance of an advantageous allele? Genetics 186: 647–668. doi:10.1534/genetics.110.119594 20660645 PMC2954473

[mcaf316-B116] Razanajatovo M, van Kleunen M, Kreft H, et al 2019. Autofertility and self-compatibility moderately benefit island colonization of plants. Global Ecology and Biogeography 28: 341–352. doi:10.1111/geb.12854

[mcaf316-B117] R Core Team . 2021. R: a language and environment for statistical computing. Vienna, Austria: R Foundation for Statistical Computing. https://www.R-project.org/

[mcaf316-B118] Reinartz JA, Les DH. 1994. Bottleneck-induced dissolution of self-incompatibility and breeding system consequences in *Aster furcatus* (Asteraceae). American Journal of Botany 81: 446–455. doi:10.1002/j.1537-2197.1994.tb15469.x

[mcaf316-B119] Rice A, Šmarda P, Novosolov M, et al 2019. The global biogeography of polyploid plants. Nature Ecology & Evolution 3: 265–273. doi:10.1038/s41559-018-0787-9 30697006

[mcaf316-B120] Riquet F, Le Cam S, Fonteneau E, Viard F. 2016. Moderate genetic drift is driven by extreme recruitment events in the invasive mollusk *Crepidula fornicata*. Heredity 117: 42–50. doi:10.1038/hdy.2016.24 27118155 PMC4901356

[mcaf316-B121] Rossetto M, Bragg J, Kilian A, McPherson H, van der Merwe M, Wilson PD. 2019. Restore and renew: a genomics-era framework for species provenance delimitation. Restoration Ecology 27: 538–548. doi:10.1111/rec.12898

[mcaf316-B122] Rossetto M, Yap J-YS, Lemmon J, et al 2021. A conservation genomics workflow to guide practical management actions. Global Ecology and Conservation 26: e01492. doi:10.1016/j.gecco.2021.e01492

[mcaf316-B123] Saunders ME, Andrew RL, Mitchell-Williams J, Pemberton P, Wandrag EM, Hunter JT. 2024. Rapid on-ground assessment after the 2019–2020 megafires reveals new information on rare and threatened plants in northern New South Wales, Australia. Austral Ecology 49: e13514. doi:10.1111/aec.13514

[mcaf316-B124] Sgrò CM, Lowe AJ, Hoffmann AA. 2011. Building evolutionary resilience for conserving biodiversity under climate change. Evolutionary Applications 4: 326–337. doi:10.1111/j.1752-4571.2010.00157.x 25567976 PMC3352557

[mcaf316-B125] Sheth SN, Morueta-Holme N, Angert AL. 2020. Determinants of geographic range size in plants. New Phytologist 226: 650–665. doi:10.1111/nph.16406 31901139

[mcaf316-B126] Shi Y-F, Shi S-H, Jiang Y-S, Liu J. 2022. A global synthesis of fire effects on soil seed banks. Global Ecology and Conservation 36: e02132. doi:10.1016/j.gecco.2022.e02132

[mcaf316-B127] Simonin KA, Roddy AB. 2018. Genome downsizing, physiological novelty, and the global dominance of flowering plants. PLoS Biology 16: e2003706. doi:10.1371/journal.pbio.2003706 29324757 PMC5764239

[mcaf316-B128] Soto Gomez M, Brown MJM, Pironon S, et al 2024. Genome size is positively correlated with extinction risk in herbaceous angiosperms. New Phytologist 243: 2470–2485. doi:10.1111/nph.19947 39080986

[mcaf316-B129] Stamatakis A . 2014. RAxML version 8: a tool for phylogenetic analysis and post-analysis of large phylogenies. Bioinformatics 30: 1312–1313. doi:10.1093/bioinformatics/btu033 24451623 PMC3998144

[mcaf316-B130] State Government of NSW and NSW Department of Climate Change, Energy, the Environment and Water . 2012. Australian soil classification (ASC) soil type map of NSW. *The Sharing and Enabling Environmental Data Portal*. https://datasets.seed.nsw.gov.au/dataset/australian-soil-classification-asc-soil-type-map-of-nsweaa10 [accessed 5 March 2025].

[mcaf316-B131] State Government of NSW and NSW Department of Climate Change, Energy, the Environment and Water . 2020. NSW state vegetation type map. *The Sharing and Enabling Environmental Data Portal*. https://datasets.seed.nsw.gov.au/dataset/nsw-state-vegetation-type-map [accessed 5 March 2025].

[mcaf316-B132] Suriyagoda LDB, Ryan MH, Gille CE, et al 2023. Phosphorus fractions in leaves. New Phytologist 237: 1122–1135. doi:10.1111/nph.18588 36328763

[mcaf316-B133] Teixeira TM, Nazareno AG. 2021. One step away from extinction: a population genomic analysis of a narrow endemic, tropical plant species. Frontiers in Plant Science 12: 730258. doi:10.3389/fpls.2021.730258 34630476 PMC8496504

[mcaf316-B134] Templeton AR . 1980. The theory of speciation via the founder principle. Genetics 94: 1011–1038. doi:10.1093/genetics/94.4.1011 6777243 PMC1214177

[mcaf316-B135] Toyama KS, Crochet P-A, Leblois R. 2020. Sampling schemes and drift can bias admixture proportions inferred by structure. Molecular Ecology Resources 20: 1769–1785. doi:10.1111/1755-0998.13234 32735380

[mcaf316-B136] Uesugi R, Nishihiro J, Tsumura Y, Washitani I. 2007. Restoration of genetic diversity from soil seed banks in a threatened aquatic plant, *Nymphoides peltata*. Conservation Genetics 8: 111–121. doi:10.1007/s10592-006-9153-6

[mcaf316-B137] Wang J . 2017. The computer program structure for assigning individuals to populations: easy to use but easier to misuse. Molecular Ecology Resources 17: 981–990. doi:10.1111/1755-0998.12650 28028941

[mcaf316-B138] Waples RS, Luikart G, Faulkner JR, Tallmon DA. 2013. Simple life-history traits explain key effective population size ratios across diverse taxa. Proceedings of the Royal Society of London B: Biological Sciences 280: 20131339. doi:10.1098/rspb.2013.1339 PMC3757969 23926150

[mcaf316-B139] Weigert A, Helm C, Meyer M, et al 2014. Illuminating the base of the annelid tree using transcriptomics. Molecular Biology and Evolution 31: 1391–1401. doi:10.1093/molbev/msu080 24567512

[mcaf316-B161] Westgate M, Kellie D, Stevenson M, Newman P. 2025. galah: Biodiversity Data from the GBIF Node Network. R package version 2.1.2. https://CRAN.Rproject.org/package=galah

[mcaf316-B140] Weir BS, Hill WG. 2002. Estimating F-statistics. Annual Review of Genetics 36: 721–750. doi:10.1146/annurev.genet.36.050802.093940 12359738

[mcaf316-B142] Whitney KD, Baack EJ, Hamrick JL, et al 2010. A role for nonadaptive processes in plant genome size evolution? Evolution; International Journal of Organic Evolution 64: 2097–2109. doi:10.1111/j.1558-5646.2010.00967.x 20148953

[mcaf316-B143] Wickham H . 2016. Ggplot2: elegant graphics for data analysis. New York: Springer.

[mcaf316-B144] Wickham H, François R, Henry L, Müller K, Vaughan D. **2023**. *Dplyr: a grammar of data manipulation*. https://cran.r-project.org/web/packages/dplyr/index.html

[mcaf316-B145] Wills TJ, Read J. 2007. Soil seed bank dynamics in post-fire heathland succession in south-eastern Australia. Plant Ecology 190: 1–12. doi:10.1007/s11258-006-9186-4

[mcaf316-B146] Wood TE, Takebayashi N, Barker MS, Mayrose I, Greenspoon PB, Rieseberg LH. 2009. The frequency of polyploid speciation in vascular plants. Proceedings of the National Academy of Sciences of the United States of America 106: 13875–13879. doi:10.1073/pnas.0811575106 19667210 PMC2728988

[mcaf316-B147] Wright GA, Schiestl FP. 2009. The evolution of floral scent: the influence of olfactory learning by insect pollinators on the honest signalling of floral rewards. Functional Ecology 23: 841–851. doi:10.1111/j.1365-2435.2009.01627.x

[mcaf316-B148] Yu G, Smith DK, Zhu H, Guan Y, Lam TT-Y. 2017. Ggtree: an r package for visualization and annotation of phylogenetic trees with their covariates and other associated data. Methods in Ecology and Evolution 8: 28–36. doi:10.1111/2041-210X.12628

[mcaf316-B149] Zaharias P, Lemoine F, Gascuel O. 2023. Robustness of Felsenstein’s versus transfer bootstrap supports with respect to taxon sampling. Systematic Biology 72: 1280–1295. doi:10.1093/sysbio/syad052 37756489 PMC10939309

