# Correction to: Geographically proximate rare species exhibit strong population divergence while maintaining intraspecific genetic diversity in *Homoranthus* (Myrtaceae)

**DOI:** 10.1093/aob/mcag185

**Published:** 2026-06-30

This is a correction to: Eilish S McMaster, Peter J Pemberton, Jeremy J Bruhl, Adam Fawcett, John T Hunter, Manu E Saunders, Elizabeth M Wandrag, Jia-Yee Samantha Yap, Ian R H Telford, Maurizio Rossetto, Rose L Andrew, Geographically proximate rare species exhibit strong population divergence while maintaining intraspecific genetic diversity in *Homoranthus* (Myrtaceae), *Annals of Botany*, Volume 138, Issue 1, 1 July 2026, Pages 101–119, https://doi.org/10.1093/aob/mcaf316

In the originally published version of the paper, the original appendix reported genome size estimates based on flow cytometry data in which the maize internal standard peaks were incorrectly assigned as 1C and 2C. These peaks are correctly identified as 2C and 4C, representing somatic nuclei in G1 and G2/M phases of the cell cycle respectively.

As a result, all genome size estimates reported in the original appendix were underestimated by a factor of two. Where specific genome size values from the appendix were cited in the main text, those values should likewise be doubled. The corrected genome size estimates are provided in the corrected appendix Table A1:

**Table A1.** Results of flow cytometry analysis for genome size estimation for Homoranthus species.

Date	Target species	Peak ID	Count^1^	Mean PE-A^2^	CV PE-A^5^	Nucleus (2C pg)^3^	Genome size (2C Mbp)^4^
28 August 2024	*Homoranthus croftianus* J.T.Hunter (Mt Tomah)	Target	1690	8282	0.1045	0.631	618
		Maize 2C	697	75027	0.0898		
		Maize 4C	1101	152117	0.0778		
28 August 2024	*Homoranthus porteri* (C.T.White) Craven & S.R.Jones (Mt Tomah)	Target	874	8370	0.1077	0.554	542
		Maize 2C	160	86442	0.0656		
		Maize 4C	539	158674	0.0849		
28 August 2024	*Homoranthus papillatus* Byrnes (Mt Tomah)	Target	1198	8306	0.1010	0.577	564
		Maize 2C	1286	82329	0.0805		
		Maize 4C	1685	163251	0.0863		
28 August 2024	*Homoranthus prolixus* Craven & S.R.Jones (Mt Tomah)	Target	602	8639	0.0981	0.609	595
		Maize 2C	425	81159	0.0789		
		Maize 4C	717	165800	0.0800		
20 August 2024	*Homoranthus flavescens* A.Cunn. ex Schauer (Mt Annan [bed 14c])	Target	3,294	26120	0.0799	0.705	690
		Maize 2C	410	211801	0.0632		
		Maize 4C	609	431687	0.0493		
20 August 2024	*Homoranthus floydii* Craven & S.R.Jones (Mt Annan [bed 10])	Target	5279	22276	0.0771	0.579	567
		Maize 2C	444	219913	0.0698		
		Maize 4C	798	446199	0.0554		
20 August 2024	*Homoranthus bebo* L.M.Copel. (Mt Annan [bed 19])	Target	5809	25971	0.0761	0.644	630
		Maize 2C	487	230683	0.0431		
		Maize 4C	1168	465419	0.0588		
20 August 2024	*Homoranthus melanostictus* Craven & S.R.Jones (Mt Annan [bed 14c])	Target	4206	26115	0.0856	0.746	729
		Maize 2C	649	200281	0.0611		
		Maize 4C	1130	405860	0.0689		
20 August 2024	*Homoranthus binghiensis J.T.Hunter* (Mt Annan [bed 217c])	Target	1945	31660	0.0568	0.699	683
		Maize 2C	194	259194	0.0532		
		Maize 4C	485	517175	0.0508		
20 August 2024	*Homoranthus lunatus* Craven & S.R.Jones (Mt Annan [bed 14c])	Target	1793	24323	0.0502	0.623	609
		Maize 2C	186	223329	0.0413		
		Maize 4C	678	447576	0.0737		
20 August 2024	*Homoranthus prolixus* Craven & S.R.Jones (Mt Annan [bed 14c])	Target	2043	26120	0.0770	0.623	609
		Maize 2C	202	239916	0.0795		
		Maize 4C	584	480614	0.0806		
20 August 2024	*Homoranthus montanus* Craven & S.R.Jones (Mt Annan [bed 14c])	Target	1782	29092	0.0597	0.639	625
		Maize 2C	400	260305	0.0824		
		Maize 4C	587	508487	0.0745		

^1^Count of nucleus observations.

^2^Mean flourescence intensity.

^3^Estimated genome size ((Target PE-A / Standard PE-A) * Standard nucleus size [pg]).

^4^Estimated nucleus size [pg] * 978 [Mbp/pg].

^5^Coefficient of variation of flourescence intensity.

instead of:

**Table A1.** Results of flow cytometry analysis for genome size estimation for *Homoranthus* species.

Date	Target species	Peak ID	Count^1^	Mean PE-A^2^	CV PE-A^5^	Nucleus (2C pg) ^3^	Genome size (2C Mbp)^4^
28 August 2024	*Homoranthus croftianus* J.T.Hunter (Mt Tomah)	Target	1690	8282	0.1045	0.300	293
		Maize 1C	697	75 027	0.0898		
		Maize 2C	1101	152 117	0.0778		
28 August 2024	*Homoranthus porteri* (C.T.White) Craven & S.R.Jones (Mt Tomah)	Target	874	8370	0.1077	0.263	257
		Maize 1C	160	86 442	0.0656		
		Maize 2C	539	158 674	0.0849		
28 August 2024	*Homoranthus papillatus* Byrnes (Mt Tomah)	Target	1198	8306	0.1010	0.274	268
		Maize 1C	1286	82 329	0.0805		
		Maize 2C	1685	163 251	0.0863		
28 August 2024	*Homoranthus prolixus* Craven & S.R.Jones (Mt Tomah)	Target	602	8639	0.0981	0.289	283
		Maize 1C	425	81 159	0.0789		
		Maize 2C	717	165 800	0.0800		
20 August 2024	*Homoranthus flavescens* A.Cunn. ex Schauer (Mt Annan [bed 14c])	Target	3294	26 120	0.0799	0.335	327
		Maize 1C	410	211 801	0.0632		
		Maize 2C	609	431 687	0.0493		
20 August 2024	*Homoranthus floydii* Craven & S.R.Jones (Mt Annan [bed 10])	Target	5279	22 276	0.0771	0.275	269
		Maize 1C	444	219 913	0.0698		
		Maize 2C	798	446 199	0.0554		
20 August 2024	*Homoranthus bebo* L.M.Copel. (Mt Annan [bed 19])	Target	5809	25 971	0.0761	0.306	299
		Maize 1C	487	230 683	0.0431		
		Maize 2C	1168	465 419	0.0588		
20 August 2024	*Homoranthus melanostictus* Craven & S.R.Jones (Mt Annan [bed 14c])	Target	4206	26 115	0.0856	0.354	346
		Maize 1C	649	200 281	0.0611		
		Maize 2C	1130	405 860	0.0689		
20 August 2024	*Homoranthus binghiensis J.T.Hunter* (Mt Annan [bed 217c])	Target	1945	31 660	0.0568	0.332	324
		Maize 1C	194	259 194	0.0532		
		Maize 2C	485	517 175	0.0508		
20 August 2024	*Homoranthus lunatus* Craven & S.R.Jones (Mt Annan [bed 14c])	Target	1793	24 323	0.0502	0.296	289
		Maize 1C	186	223 329	0.0413		
		Maize 2C	678	447 576	0.0737		
20 August 2024	*Homoranthus prolixus* Craven & S.R.Jones (Mt Annan [bed 14c])	Target	2043	26 120	0.0770	0.296	289
		Maize 1C	202	239 916	0.0795		
		Maize 2C	584	480 614	0.0806		
20 August 2024	*Homoranthus montanus* Craven & S.R.Jones (Mt Annan [bed 14c])	Target	1782	29 092	0.0597	0.303	297
		Maize 1C	400	260 305	0.0824		
		Maize 2C	587	508 487	0.0745		

^1^Count of nucleus observations.

^2^Mean flourescence intensity.

^3^Estimated genome size ((Target PE-A / Standard PE-A) * Standard nucleus size [pg]).

^4^Estimated nucleus size [pg] * 978 [Mbp/pg].

^5^Coefficient of variation of flourescence intensity.

The conclusions and interpretation of the main text are unaffected by this correction.

