# Supplementary material for: Geographically proximate rare species exhibit strong population divergence while maintaining intraspecific genetic diversity in Homoranthus (Myrtaceae)
Source: Ann Bot. 2025 Dec 9;138(1):101–19. doi: 10.1093/aob/mcaf316 (PMC13409159; doi:10.1093/aob/mcaf316)
Supplement: mcaf316_Supplementary_Data [file mcaf316_supplementary_data.docx]

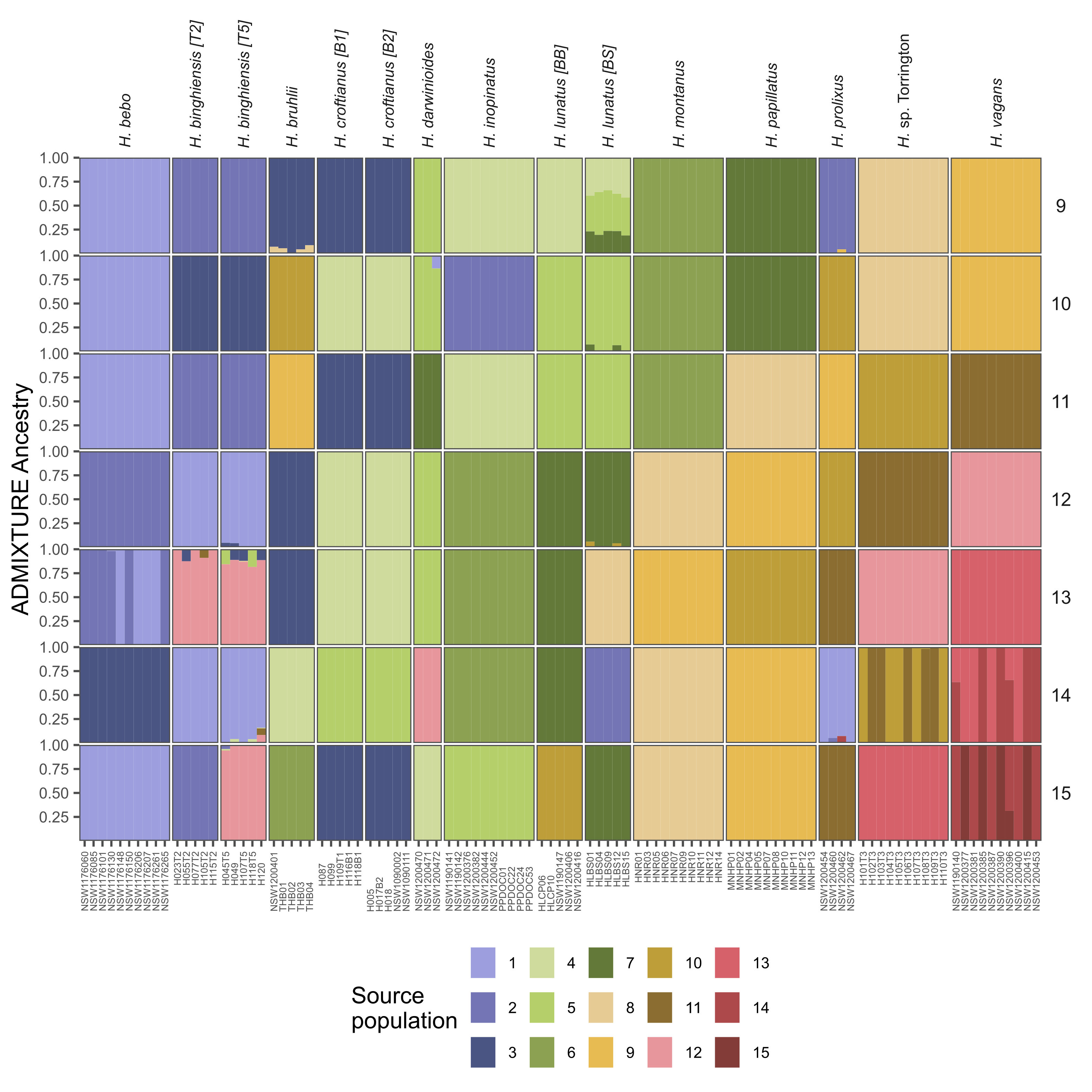

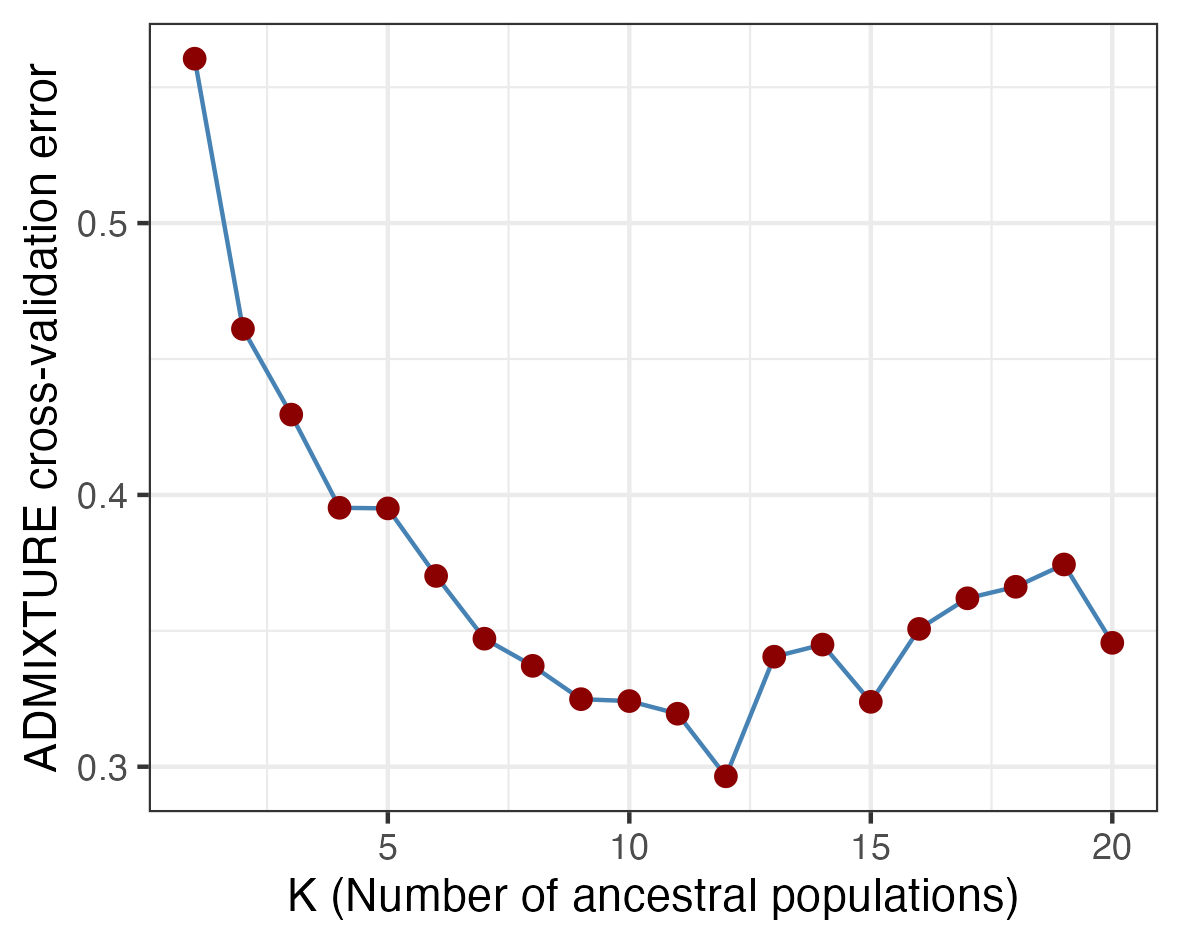
 **Figure S1.** Cross validation error from unsupervised ADMIXTURE analysis. The minimum CV error value is K=12, indicating that 12 ancestral populations is optimal.

**Figure S2.** ADMIXTURE ancestry estimation from unsupervised analysis from K=9 to K=15. Species groups have been subset to have a maximum of 10 samples to reduce bias from uneven sample sizes.

| **Species** | **Site** | **loci** | **n** | **Ho** | **He** | **uHe** | **Fis** | **Fst** | **uFis** | **f.2.5%** | **f.97.5%** | **f.μ** | **he.2.5%** | **he.97.5%** | **he.μ** | **ho.2.5%** | **ho.97.5%** | **ho.μ** | **HWE.p** |
| --- | --- | --- | --- | --- | --- | --- | --- | --- | --- | --- | --- | --- | --- | --- | --- | --- | --- | --- | --- |
| *H. bebo* | Camp Creek East | 2644 | 45 | 0.223 | 0.343 | 0.347 | 0.349 | 0.004 | 0.357 | 0.353 | 0.373 | 0.363 | 0.338 | 0.348 | 0.343 | 0.213 | 0.223 | 0.218 | 0.00 |
| *H. bebo* | Camp Creek West | 2644 | 8 | 0.207 | 0.297 | 0.320 | 0.303 | 0.139 | 0.355 | 0.266 | 0.304 | 0.285 | 0.289 | 0.303 | 0.296 | 0.195 | 0.209 | 0.202 | 1.00 |
| *H. binghiensis* | Torrington Site 2 | 2737 | 22 | 0.137 | 0.271 | 0.280 | 0.496 | 0.121 | 0.511 | 0.460 | 0.488 | 0.474 | 0.265 | 0.278 | 0.272 | 0.128 | 0.136 | 0.132 | 0.00 |
| *H. binghiensis* | Torrington Site 5 | 2737 | 25 | 0.173 | 0.269 | 0.276 | 0.359 | 0.127 | 0.374 | 0.338 | 0.364 | 0.351 | 0.263 | 0.276 | 0.269 | 0.163 | 0.174 | 0.168 | 0.00 |
| *H. croftianus* | Bolivia Site 1 | 2312 | 28 | 0.172 | 0.294 | 0.300 | 0.416 | 0.151 | 0.428 | 0.390 | 0.417 | 0.403 | 0.287 | 0.300 | 0.294 | 0.161 | 0.171 | 0.166 | 0.00 |
| *H. croftianus* | Bolivia Site 2 | 2312 | 39 | 0.185 | 0.321 | 0.326 | 0.425 | 0.072 | 0.434 | 0.414 | 0.436 | 0.424 | 0.315 | 0.327 | 0.321 | 0.176 | 0.185 | 0.180 | 0.00 |
| *H. inopinatus* | Ballandean | 1828 | 16 | 0.235 | 0.351 | 0.364 | 0.332 | 0.000 | 0.356 | 0.316 | 0.349 | 0.332 | 0.345 | 0.357 | 0.351 | 0.225 | 0.239 | 0.232 | 0.00 |
| *H. lunatus* | Basket Swamp | 2044 | 9 | 0.136 | 0.230 | 0.248 | 0.409 | 0.318 | 0.451 | 0.348 | 0.396 | 0.372 | 0.222 | 0.239 | 0.230 | 0.128 | 0.142 | 0.134 | 1.00 |
| *H. lunatus* | BB Cypress | 2044 | 9 | 0.188 | 0.239 | 0.257 | 0.213 | 0.292 | 0.266 | 0.155 | 0.196 | 0.176 | 0.231 | 0.247 | 0.239 | 0.181 | 0.198 | 0.189 | 1.00 |
| *H. lunatus* | BB Morgan | 2044 | 14 | 0.143 | 0.242 | 0.253 | 0.409 | 0.285 | 0.435 | 0.363 | 0.402 | 0.383 | 0.233 | 0.250 | 0.241 | 0.134 | 0.147 | 0.140 | 0.00 |
| *H. montanus* | Hillview NR | 1847 | 13 | 0.251 | 0.349 | 0.365 | 0.281 | 0.000 | 0.312 | 0.268 | 0.305 | 0.288 | 0.344 | 0.355 | 0.349 | 0.241 | 0.255 | 0.248 | 0.00 |
| *H. papillatus* | Mt Norman | 2565 | 11 | 0.217 | 0.327 | 0.345 | 0.336 | 0.000 | 0.370 | 0.282 | 0.313 | 0.297 | 0.322 | 0.332 | 0.327 | 0.207 | 0.219 | 0.213 | 0.00 |
| *H. prolixus* | Ironbark | 1378 | 4 | 0.223 | 0.377 | 0.440 | 0.410 | 0.000 | 0.494 | 0.308 | 0.367 | 0.337 | 0.372 | 0.382 | 0.377 | 0.211 | 0.232 | 0.222 | 1.00 |
| *H*. sp. Torrington | Torrington Site 3 | 2379 | 10 | 0.188 | 0.306 | 0.325 | 0.386 | 0.000 | 0.422 | 0.297 | 0.330 | 0.314 | 0.300 | 0.312 | 0.306 | 0.180 | 0.191 | 0.185 | 0.00 |
| *H. vagans* | Wundal Range NP | 2143 | 15 | 0.191 | 0.346 | 0.360 | 0.447 | 0.000 | 0.469 | 0.451 | 0.485 | 0.468 | 0.341 | 0.352 | 0.346 | 0.181 | 0.193 | 0.187 | 0.00 |

**Table S1.** Comprehensive genetic diversity statistics for *Homoranthus* species across collection sites. The table includes the number of loci passing filters (minor allele frequency ≥5%, maximum 30% missing data per species), number of individuals sampled (n), observed heterozygosity (Ho), expected heterozygosity (He) and its unbiased version (uHe), inbreeding coefficients (Fis, uFis), and Weir & Cockerham’s (1984) Fst [(Ht–He)/Ht]. Mean and 95% confidence intervals of Ho, He, and Fis were calculated from 1,000 bootstrap replicates. Hardy–Weinberg exact test P-values (HWE.p) are provided.

**Table S2.** Extent of occurrence (EOO) and area of occupancy (AOO) in km^2^, and their percentile ranks among NSW plant species (Le Breton *et al.* 2019) for the study species of *Homoranthus*.

| **Species** | **EOO** | **AOO** | **EOO percentile** | **AOO percentile** |
| --- | --- | --- | --- | --- |
| *H. bebo* | 1.72 | 12 | 0.3 | 1.6 |
| *H. binghiensis* | 68.02 | 52 | 1.0 | 7.4 |
| *H. bruhlii* | 0.04 | 8 | 0.3 | 0.9 |
| *H. croftianus* | 0.52 | 8 | 0.3 | 0.9 |
| *H. darwinioides* | 4178.20 | 68 | 5.9 | 9.6 |
| *H. inopinatus* | 0.56 | 8 | 0.3 | 0.9 |
| *H. lunatus* | 7.42 | 12 | 0.4 | 1.6 |
| *H. melanostictus* | 51855.99 | 88 | 18.7 | 13.1 |
| *H. montanus* | 6.45 | 16 | 0.4 | 2.0 |
| *H. papillatus* | 0.37 | 8 | 0.3 | 0.9 |
| *H. prolixus* | 8375.01 | 140 | 8.3 | 20.7 |
| *H.* sp. Torrington | 0.84 | 12 | 0.3 | 1.6 |
| *H. vagans* | 4.58 | 16 | 0.4 | 2.0 |
